# Supplementary material for: Perceived feasibility, facilitators and barriers to incorporating point-of-care testing for SARS-CoV-2 into emergency medical services by ambulance service staff: a survey-based approach
Source: BMJ Open. 2022 Nov 3;12(11):e064038. doi: 10.1136/bmjopen-2022-064038 (PMC9638752; doi:10.1136/bmjopen-2022-064038)
Supplement: Supplementary data [file bmjopen-2022-064038supp001.pdf]

Supplementary Material 1: Survey 1 questions

Page 1: Landing page

COVID-19 rapid testing within the Ambulance Service

Dear Participant,

The aim of this survey is to understand the potential role and perceived value of rapid testing within the ambulance service.

You have been invited to participate because you are a health or social care professional in UK.

The survey should take no more than 3 minutes to complete.

No personal or sensitive data is required, and your response is anonymous and will be kept confidential.

Results will be used to inform future research in this area.

If you have any questions or would like to get in touch please do not hesitate to contact: nihr.newcastle.mic@ncl.ac.uk.

By continuing to the next page you will be consenting to participate in this survey.

Thank you for taking part.

Page 2: Participant Information

1. **Which region of the UK do you work in?**

   Scotland

   Northern Ireland

   Wales

   North West England

   North East England

   Yorkshire & The Humber

   West Midlands

   East Midlands

   South West

   South East

   East of England

   Greater London

2. **What is your current job role?**

   Ambulance Service Team – Ambulance care assistant and Patient Transport Service (PTS) driver

   Ambulance Service Team – Call handler/emergency medical dispatcher

   Ambulance Service Team – Emergency care assistant

   Ambulance Service Team – Emergency medical technician

   Ambulance Service Team - Paramedic

   Ambulance Service Team – Experience paramedic

Ambulance Service Team – Patient Transport Service (PTS) call handler

Ambulance Service Team – Integrated urgent care/NHS 111

3. **Please use this space to add any other information regarding your role, if applicable (eg. specialism, additional expertise, area of interest)**

    [Free Text]

Page 3: COVID-19 rapid testing within the Ambulance Service

4. **Do you already conduct any tests for respiratory infection in a pre-hospital setting? If yes, please describe the test(s) below:**

    [Free text]

5. **How feasible do you think it would be to test patients for COVID-19 before transfer to hospital?**

    Very high

    High

    Neutral

    Low

    Very Low

    a. **Please add any additional comments on feasibility here:**

    [Free text]

6. **What would be the optimal or ideal sampling technique for a COVID-19 test in an ambulance setting?**

    Nasopharyngeal swab

    Finger prick blood test

    Sputum sample

    Saliva sample

    Other (please specify)

    [Free text]

7. **How helpful do you feel the deployment of rapid COVID-19 testing in an ambulance setting would be?**

    Very helpful

    Quite helpful

    Neither helpful nor unhelpful

    Quite unhelpful

    Very unhelpful

8. **What do you think the main use case for rapid COVID-19 testing in ambulances would be?**

Triaging of patients prior to arrival at secondary care facilities to improve handover and flow

Risk stratification of patients e.g. can a patient be safely left at home

Aid decision making on where the patient should be referred to next e.g. Hot-Hub

Rationalising PPE use for ambulance service staff

Aiding decision to bring additional crew to a call or not

Other (please specify)

[Free Text]

9. What do you think the most important characteristics of a test on board ambulances would be?

Time to result – Most important – very important – important – less important – not important

Accuracy – Most important – very important – important – less important – not important

Ease of use – Most important – very important – important – less important – not important

Robustness – Most important – very important – important – less important – not important

Size of the kit – Most important – very important – important – less important – not important

Storage of the kit – Most important – very important – important – less important – not important

Portability of the kit – Most important – very important – important – less important – not important

Other (please specify) – Most important – very important – important – less important – not important

10. What would be the maximum acceptable time to results (TTR) for an ambulance-based COVID test?

[Slider scale 'Time to results in minutes', Minimum value = 0, Maximum value = 45]

11. What is the minimum sensitivity that a test in ambulances should have?

>60% (at least 6/10 people with COVID will get a positive result)

>70% (at least 7/10 people with COVID will get a positive result)

>80% (at least 8/10 people with COVID will get a positive result)

>90% (at least 9/10 people with COVID will get a positive result)

12. What is the minimum specificity that a test in ambulances should have?

>60% (at least 6/10 people without COVID will get a negative result)

>70% (at least 7/10 people without COVID will get a negative result)

>80% (at least 8/10 people without COVID will get a negative result)

>90% (at least 9/10 people without COVID will get a negative result)

13. What do you think the main concerns of adding a test on board an ambulance would be?

[Free Text]

14. Is there anything else you feel is important to tell us about testing in ambulances?

[Free Text]

15. If you would be willing to speak to us further about COVID-19 testing within the ambulance service, please provide details below

[Name]

[Preferred Email Address]
