# Supplementary material for: Perceived feasibility, facilitators and barriers to incorporating point-of-care testing for SARS-CoV-2 into emergency medical services by ambulance service staff: a survey-based approach
Source: BMJ Open. 2022 Nov 3;12(11):e064038. doi: 10.1136/bmjopen-2022-064038 (PMC9638752; doi:10.1136/bmjopen-2022-064038)
Supplement: Supplementary data [file bmjopen-2022-064038supp002.pdf]

Supplementary Material 2: Survey 2 questions

Page 1: Landing page

Use cases and diagnostic profiles for COVID-19 rapid testing within the Ambulance Service

Dear Participant,

The aim of this survey is to further understand the potential role for COVID-19 diagnostic tests in different scenarios relevant to the ambulance service.

You have been invited to participate because you are a health or social care professional in UK and have previously provided contact details for us to speak to you further about COVID-19 testing in an ambulance setting.

The follow up survey should take no more than 3 minutes to complete.

No personal or sensitive data is required, and your response is anonymous and will be kept confidential.

Results will be used to inform future research in this area.

If you have any questions or would like to get in touch please do not hesitate to contact: nihr.newcastle.mic@ncl.ac.uk.

By continuing to the next page you will be consenting to participate in this survey.

Thank you for taking part.

Page 2: Use cases and diagnostic profiles for COVID-19 rapid testing within the Ambulance Service

The results of our initial survey highlighted a number of potential use cases that participants felt may be important. In this follow up series of questions we would like to better understand the qualities and characteristics of COVID-19 tests that you feel are necessary for each use case.

Please rate how useful you feel each type of test would be for each of the use cases from 'Extremely useful' to 'Not at all useful'

Sensitivity - The ability of a test to correctly identify a patient with the disease.
[The test is positive when the patient has COVID-19]

Specificity - The ability of a test to correctly identify a patient who does not have the disease.
[The test is negative when the patient does not have COVID-19]

1. **A test with greater than 95% sensitivity and specificity, less than 15 minutes time to results**
   a. Aid decision making on where the patient should be referred to next e.g. Hot-Hub

      Extremely useful – Very useful – Somewhat useful – Not so useful – Not at all useful

   b. Risk stratification of patients e.g. can a patient be safely left at home

      Extremely useful – Very useful – Somewhat useful – Not so useful – Not at all useful

   c. Triaging of patients prior to arrival at secondary care facilities to improve handover and flow

      Extremely useful – Very useful – Somewhat useful – Not so useful – Not at all useful

   d. Rationalising PPE use for ambulance service staff

      Extremely useful – Very useful – Somewhat useful – Not so useful – Not at all useful

   - Please provide any additional comments or reasoning here:

     [Free Text]

2. **A test with greater than 80% sensitivity and specificity, less than 15 minutes time to results**
   a. Aid decision making on where the patient should be referred to next e.g. Hot-Hub

Extremely useful – Very useful – Somewhat useful – Not so useful – Not at all useful

b. Risk stratification of patients e.g. can a patient be safely left at home

Extremely useful – Very useful – Somewhat useful – Not so useful – Not at all useful

c. Triaging of patients prior to arrival at secondary care facilities to improve handover and flow

Extremely useful – Very useful – Somewhat useful – Not so useful – Not at all useful

d. Rationalising PPE use for ambulance service staff

Extremely useful – Very useful – Somewhat useful – Not so useful – Not at all useful

- Please provide any additional comments or reasoning here:

  [Free Text]

3. A test with less than 80% sensitivity and specificity, less than 15 minutes time to results

a. Aid decision making on where the patient should be referred to next e.g. Hot-Hub

Extremely useful – Very useful – Somewhat useful – Not so useful – Not at all useful

b. Risk stratification of patients e.g. can a patient be safely left at home

Extremely useful – Very useful – Somewhat useful – Not so useful – Not at all useful

c. Triaging of patients prior to arrival at secondary care facilities to improve handover and flow

Extremely useful – Very useful – Somewhat useful – Not so useful – Not at all useful

d. Rationalising PPE use for ambulance service staff

Extremely useful – Very useful – Somewhat useful – Not so useful – Not at all useful

- Please provide any additional comments or reasoning here:

  [Free Text]
