# Supplementary material for: Perceived feasibility, facilitators and barriers to incorporating point-of-care testing for SARS-CoV-2 into emergency medical services by ambulance service staff: a survey-based approach
Source: BMJ Open. 2022 Nov 3;12(11):e064038. doi: 10.1136/bmjopen-2022-064038 (PMC9638752; doi:10.1136/bmjopen-2022-064038)
Supplement: Author's manuscript [file bmjopen-2022-064038.draft_revisions.pdf]

BMJ Open

BMJ Open is committed to open peer review. As part of this commitment we make the peer review history of every article we publish publicly available.

When an article is published we post the peer reviewers' comments and the authors' responses online. We also post the versions of the paper that were used during peer review. These are the versions that the peer review comments apply to.

The versions of the paper that follow are the versions that were submitted during the peer review process. They are not the versions of record or the final published versions. They should not be cited or distributed as the published version of this manuscript.

BMJ Open is an open access journal and the full, final, typeset and author-corrected version of record of the manuscript is available on our site with no access controls, subscription charges or pay-per-view fees (http://bmjopen.bmj.com).

If you have any questions on BMJ Open's open peer review process please email info.bmjopen@bmj.com

The perceived feasibility, facilitators and barriers to incorporating point-of-care testing for SARS-CoV-2 into Emergency Medical Services by Ambulance Service staff: A survey-based approach

Journal:	*BMJ Open*
Manuscript ID	bmjopen-2022-064038
Article Type:	Original research
Date Submitted by the Author:	21-Apr-2022

Complete List of Authors:	Green, Kile; Newcastle University, NIHR Newcastle In Vitro Diagnostics Co-operative, Translational and Clinical Research Institute<br>Micocci, Massimo; Imperial College London, NIHR London In Vitro Diagnostics Co-operative<br>Hicks, Timothy; Newcastle Upon Tyne Hospitals NHS Foundation Trust; Newcastle University, NIHR Newcastle In Vitro Diagnostics Co-operative, Translational and Clinical Research Institute<br>Winter, Amanda; Newcastle Upon Tyne Hospitals NHS Foundation Trust; Newcastle University, NIHR Newcastle In Vitro Diagnostics Co-operative, Translational and Clinical Research Institute<br>Martin, Joanne E; Barts and The London NHS Trust, Centre for Genomics and Child Health, Blizard Institute,<br>Shinkins, Bethany; University of Leeds, Academic Unit of Health Economics<br>Shaw, Lisa; Newcastle University, Stroke Research Group, Population Health Sciences,<br>Price, Christopher; Newcastle University, Institute of Neuroscience,<br>Davies, Kerrie; University of Leeds, Healthcare Associated Infections Research Group<br>Allen, A; Newcastle University, NIHR Newcastle In Vitro Diagnostics Co-operative, Translational and Clinical Research Institute; Newcastle Upon Tyne Hospitals NHS Foundation Trust

Keywords:	COVID-19, QUALITATIVE RESEARCH, Molecular diagnostics < INFECTIOUS DISEASES

SCHOLARONE™ Manuscripts

I, the Submitting Author has the right to grant and does grant on behalf of all authors of the Work (as defined in the below author licence), an exclusive licence and/or a non-exclusive licence for contributions from authors who are: i) UK Crown employees; ii) where BMJ has agreed a CC-BY licence shall apply, and/or iii) in accordance with the terms applicable for US Federal Government officers or employees acting as part of their official duties; on a worldwide, perpetual, irrevocable, royalty-free basis to BMJ Publishing Group Ltd ("BMJ") its licensees and where the relevant Journal is co-owned by BMJ to the co-owners of the Journal, to publish the Work in this journal and any other BMJ products and to exploit all rights, as set out in our licence.

The Submitting Author accepts and understands that any supply made under these terms is made by BMJ to the Submitting Author unless you are acting as an employee on behalf of your employer or a postgraduate student of an affiliated institution which is paying any applicable article publishing charge ("APC") for Open Access articles. Where the Submitting Author wishes to make the Work available on an Open Access basis (and intends to pay the relevant APC), the terms of reuse of such Open Access shall be governed by a Creative Commons licence – details of these licences and which Creative Commons licence will apply to this Work are set out in our licence referred to above.

Other than as permitted in any relevant BMJ Author's Self Archiving Policies, I confirm this Work has not been accepted for publication elsewhere, is not being considered for publication elsewhere and does not duplicate material already published. I confirm all authors consent to publication of this Work and authorise the granting of this licence.

For peer review only

The perceived feasibility, facilitators and barriers to incorporating point-of-care testing for SARS-CoV-2 into Emergency Medical Services by Ambulance Service staff: A survey-based approach

Dr Kile Green*[1], Dr Massimo Micocci[2], Mr Timothy Hicks[1], Ms Amanda Winter[1], Prof Joanne E Martin[3], Dr Bethany Shinkins[4], Dr Lisa Shaw[5], Professor Christopher I. Price[5], Dr Kerrie Davies[6,7], Dr A. Joy Allen[1] on behalf of the CONDOR Steering group

1: NIHR Newcastle In Vitro Diagnostics Co-operative, Newcastle-upon-Tyne Hospitals Foundation Trust, Newcastle, United Kingdom.

2: NIHR London In Vitro Diagnostics Co-operative, Imperial College London, London, United Kingdom

3: Centre for Genomics and Child Health, Blizard Institute, Barts and The London School of Medicine and Dentistry, Queen Mary, University of London, London, United Kingdom.

4: Test Evaluation Group, Leeds Institute for Health Sciences, University of Leeds, Leeds, UK

5: Stroke Research Group, Population Health Sciences Institute, Newcastle University, UK.

6: Healthcare Associated Infections Research Group, University of Leeds and Leeds Teaching Hospitals NHS Trust, Leeds, UK

7: NIHR Leeds In Vitro Diagnostics Co-operative, Leeds Teaching Hospitals NHS Trust, Leeds, UK

** Corresponding author [Dr Kile Green, kile.green@newcastle.ac.uk]*

Abstract

Objectives: This body of work aimed to elicit ambulance service staff's opinions on the barriers and facilitators to adoption, and clinical utility of incorporating rapid SARS-CoV-2 testing during ambulance assessments.

Setting: Emergency and non-emergency care ambulatory services in the UK were approached to take part.

Participants: Current, practicing members of the UK ambulance service (frontline, call-handlers and management staff) were included in this body of work.

Results: Survey 1: 226 responses were collected between 3rd December 2020 and 11th January 2021, 179 (79.2%) of which were completed in full. While the majority of respondents indicated that an ambulance-based testing strategy was feasible in concept (143/190, 75.3%), major barriers to adoption were noted. Many open-ended responses cited concerns regarding misuse of the service by the general public and other healthcare services, timing and conveyance issues, and increased workloads, alongside training and safety concerns. Survey 2: 26 responses were received between 8th February 2021 and 22nd February 2021 to this follow-up survey. Building on the responses from survey 1, survey 2 revealed conveyance

For peer review only - http://bmjopen.bmj.com/site/about/guidelines.xhtml

decision-making and risk stratification to be the most frequently prioritised use cases amongst ambulance service staff. Optimal test characteristics for adoption of a test according to respondents were; accuracy (above 90% sensitivity and specificity), rapidity (<30 minutes time to results) and ease of sample acquisition. These characteristics were deemed most important for feasibility of use in this setting.

Conclusions: The majority of commercially available lateral flow devices are unlikely to be supported by paramedics as their duty of care requires both rapid and accurate results that can inform clinical decision making in an emergency situation. Further investigation is needed to define acceptable test characteristics and criteria required for ambulance service staff to be confident and supportive of deployment of a SARS-CoV-2 test in an emergency care setting.

Keywords: Ambulance Service, COVID-19, feasibility, point-of-care testing, SARS-CoV-2, survey, thematic analysis.

For peer review only

Background

In response to the COVID-19 pandemic, there was large scale production of rapid, portable clinical tests for SARS-CoV-2 with CE-IVD approval [1, 2]. Like any test, their true value hinges on their role within a clinical pathway, the real-world accuracy of the test within that role, and the efficacy of the decisions made following test results [3, 4]. There has been significant debate about whether some of these tests are 'fit for purpose', for example in the UK, the widespread use of rapid antigen-based lateral flow devices (LFDs) has raised concerns because their sensitivity has been shown to drop dramatically (from 73% to 48.8%) when carried out by non-trained healthcare workers [5-7].

In an attempt to provide greater clarity on the minimum acceptable and desirable characteristics for rapid, point-of-care (PoC) tests for SARS-CoV-2, the World Health Organisation (WHO) and the UK Medicines and Healthcare products Regulatory Agency (MHRA) developed and published target product profiles (TPPs) for novel tests in this space [8]. However, given the vast number of use cases for PoC tests for SARS-CoV-2 and the ongoing need to provide guidance throughout the UK pandemic, the TPPs were reasonably generic. Further work is required to understand the exact role of these tests within specific settings and the potential downstream consequences of testing [9].

Ambulance services have previously been highlighted as potential settings where rapid PoC testing, including for SARS-CoV-2, could add value for patients and the healthcare system, as they are frequently the first point of contact with healthcare services during an emergency [10-12]. However, due to the unique environment, including reliance upon a dispersed workforce, operating under time pressures and with limited equipment [13], it is necessary to identify setting-specific clinical scenarios where testing could effectively integrate into ambulance care processes. At the time of survey dissemination, at the height of the COVID-19 lockdown in the UK, some ambulance service trusts had begun to trial the use of PoC SARS-CoV-2 testing, but this was not widely performed. The objective of this body of work was to generate evidence and support decision making on the integration of SARS-CoV-2 POC tests using a mixed-methodology approach to elicit views from ambulance service staff about factors that would facilitate or act as a barrier to PoC testing for SARS-CoV-2 in this setting. Collecting feedback and insights from end users of POC devices would enable decision makers to determine the feasibility and expected use cases in an ambulance setting, and work to mitigate any potential barriers to adoption to ensure the greatest added value to healthcare is achieved.

Methods

Patient and public involvement

For peer review only

The NIHR Newcastle In Vitro Diagnostics Co-operative (MIC) Insight PPIE Panel were consulted at various stages of the project. Initial comments were gathered on questions exploring COVID-19 testing and interpretation of results. Their feedback helped to formulate and refine questions to better facilitate participant understanding. The PPIE leads wrote a lay summary of the project outputs for dissemination via the MIC website and social media.

Study design

This study consisted of two surveys, each following a mixed-methodology approach, designed to capture opinions on the perceived feasibility and prioritisation of potential clinical scenarios where PoC SARS-CoV-2 tests could add value to healthcare. The surveys were developed by test evaluation methodologists (KG, TH, AW, MM and AJA), who are all experienced in qualitative methods for diagnostic evaluation, and were constructed using the online survey tool SurveyMonkey [14]. No questions were mandatory on either survey. No sensitive or patient identifiable data were included.

Respondents to Survey 1 were invited to complete Survey 2.

- Survey 1 [Supplemental materials 1] was launched on 3rd December 2020 and was open to responses until 11th January 2021. The reach of the survey is unknown due to the multiple methods of dissemination, however 212 ambulance service respondents started the survey. Survey 1 aimed to identify key facilitators and barriers to the adoption of PoC SARS-CoV-2 tests in ambulances, with a focus on prioritising the key clinical scenarios where the tests could add value alongside the practical aspects that could affect adoption (environmental factors, test turnaround, usability).
  Survey 2 (follow-up) [Supplemental materials 2] was disseminated to interested parties on 8th February 2021 and was open to responses until 22nd February 2021. 50 respondents to Survey 1 were sent Survey 2 via email. 26/50 (52%) respondents completed Survey 2. This survey aimed to identify the most acceptable diagnostic accuracy requirements of rapid tests specifically to meet the prioritised clinical scenarios that were identified in Survey 1.

Setting

The surveys included in this body of work were conducted at the height of the UK pandemic lockdown period when daily cases were at the highest levels seen in the UK since wide-scale reporting began[15]. Some NHS hospital Trusts reported 100% capacity of critical care beds at this time and the matter was discussed extensively in media and during parliamentary sessions[16]. It is important to highlight this context in mind as it reflects the urgency at which the work was performed and affected the capacity for participants to respond to the survey, the content of the survey responses and the concerns raised at this time.

Dissemination & participant selection

For peer review only

Participants were selected by purposive sampling with additional snowballing, targeting ambulance service staff at all levels to gain breadth of viewpoints across the service The primary questionnaire was disseminated via email by Ambulance Trust Research and Development (R&D) teams as well as through promotion of the survey via email (JM) to professionals involved in rapid testing in the acute sector. Survey 2 was disseminated via email directly to those respondents to Survey 1 who had stated that they would be interested in providing additional input through follow-up questionnaire. Participation was voluntary and anonymised.

Purpose & Structure

Survey 1 [Supplemental materials 1] was structured with a mixture of multiple choice and free text answers to capture the following:

1) Demographics, exposure/familiarity with SARS-CoV-2 tests.
2) Desired test characteristics in general (including accuracy, ease of use, time to result (TTR)).
3) Potential clinical scenarios where PoC testing for SARS-CoV-2 testing could add value
4) Feasibility of integrating PoC tests into current processes and patient pathways.

Survey 1 included two open-ended questions: 'What do you think the main concerns of adding a test on board the ambulance would be?' and 'Is there anything else you feel is important to tell us about testing in ambulances?'. In survey 2 [Supplemental materials 2], respondents were asked to state whether three hypothetical tests with sensitivities and specificities of >95%, >80% and <80% respectively would be useful for each of the priority clinical scenarios identified in Survey 1 (on a scale from 'Extremely useful' to 'Not useful at all').

To ensure respondents understood complex technical performance terminology such as sensitivity and specificity, multiple choice answers included an example description of the value, e.g. 60% sensitivity (6/10 people with COVID-19 get a positive test result).

Reporting guidelines

The Standards for Reporting Qualitative Research (SRQR) checklist were used to structure the methodology and results of this study alongside the 'CHERRIES' checklist for reporting of online surveys [17, 18].

Data analysis

Quantitative analysis of structured responses Data processing and generation of summary statistics was performed using Microsoft Excel. As all questions were optional, denominators for each question reflect the number of responses to that individual question. Graphics and visuals were produced using the 'CANVA' web-tool suite to display the aggregated results. For questions using a Likert scale, a mean summary score (with standard deviation) was

For peer review only

calculated based on the following mapping - Most Important = 2, Very Important = 1, Important = 0, Less Important = -1, Not Important = -2. Final scores were then ranked from highest to lowest.

Thematic analysis of free text responses A Framework Analysis Method[19] was used to organise data from the qualitative, open-ended questions. Thematic analysis aimed to identify common issues and concerns about testing within the ambulance service. Initial coding of the responses were performed independently by two researchers (KG and MM), with a third acting as adjudicator (AJA) to reduce bias and improve consistency. Codes were discussed internally to reach an agreement on definitions, content and to improve validity. Codes were then clustered under four main categories to identify cross-cutting patterns. Final themes and definitions were agreed collaboratively amongst the qualitative researchers.

Results:

Demographics

Of the 212 ambulance service respondents, 150 were listed as ‘Paramedics’ with 55 of those considered ‘Experienced paramedics’ – with more than 2 years of experience in the role. Other roles included technical, support and call-handling staff.

One hundred and nineteen (56.1%) of the ambulance staff respondents (hereafter referred to as ‘respondents’) were based in the east of England, with a further 49 (23.1%) based in the Greater London region (Table 1). All regions of England and Northern Ireland were represented by at least one respondent.

Table 1: Summary table of Ambulance service responders by region.

Region	Ambulance service staff
**East Midlands**	5
**East of England**	114
**Greater London**	46
**North East**	2
**North West**	3
**Northern Ireland**	1
**South East**	13
**South West**	8
**West Midlands**	5
**Yorkshire & the Humber**	15
**Total**	**212**

Feasibility of testing

The majority of respondents answering questions on feasibility stated that it would be feasible to test patients for SARS-CoV-2 before transfer to hospital with 143/190 (75.3%) stating ‘High’ or ‘Very high’ feasibility, including 22/26 (84.6%) of the respondents who had

For peer review only

previously noted prior experience of using lateral flow devices (LFDs) for SARS-CoV-2 or other indications.

Twenty six out of 101 (24.7%) stated they had experience conducting tests for *any* respiratory infection in a pre-hospital setting, including (LFDs) and oxygen saturation monitors. Additionally, blood glucose monitoring tests were already in use and routinely performed on route to secondary care.

Use cases and sampling method

The top four ranked clinical scenarios where a PoC test for SARS-CoV-2 was deemed to add value, accounted for 92.4% of the total responses to this question [Figure 1]:

1) *'Triaging of patients prior to arrival at secondary care facilities to improve handover'* (83/170, 48.8%);
2) *'Aid decision making on where a patient should be referred to next'* (40/170, 23.5%);
3) *'Risk stratification of patients to determine if a patient can be safety left at home'* (28/170, 16.5%);
4) *'Rationalising PPE use for ambulance service staff'* (6/170, 3.5%).

Other responses were free-text answers including 'all options', 'none of the options' and other niche uses.

Irrespective of other technology considerations, finger prick blood sampling was stated as the optimal sampling technique by one third of the respondents (125/189, 66.1%). Twenty nine (29/189, 15.3%) and 19 (19/189, 10.1%) respondents preferred nasopharyngeal swabbing and saliva sampling, respectively. Respondents using the 'other' option were largely in favour of testing, regardless of the specific method used.

Importance of test characteristics

'Accuracy' and 'Time to result' of the test were the most important characteristics to the responders with a mean adjusted score of 1.47 (std.dev 0.44) and 1.32 (std.dev 0.35), respectively [Figure 2]. 'Storage of the kit' (mean score 0.14) and 'size of the kit' (mean score -0.13) were amongst the least important characteristics. Additional comments provided by paramedics included 'minimisation of staff exposure time', 'room temperature storage' and 'cost'.

Time to results 88/170 respondents (51.8%) stated a maximum acceptable TTR of 15 minutes or less, with 162/170 (95.3%) selecting 30 minutes or less.

Sensitivity & Specificity The majority of responses required over 80% sensitivity (56/170, 32.9% selected 'above 80% sensitivity', 73/170, 42.9% of responders selected 'above 90% sensitivity'). Similar responses were obtained for minimum specificity requirements (43/170, 24.7% respondents selected 'above 80% specificity', and 79/170, 46.5% selected 'above 90% specificity').

Thematic analysis of qualitative responses A total of 11 subthemes were generated from the 128 responses to the two free-text questions from the primary survey (Survey 1) and

237 categorised into 4 broad themes (Service level implications, Direct impact on patient care, Practical use of the test and Safety) to identify cross-cutting patterns. Subtheme definitions have been includes in table format [Table 2]. Representative quotes in the results have been assigned a numerical identification code (IC) based on [participant number.

241 **Table 2: Summary table of themes for thematic analysis of open-ended responses.**

THEME	SUB-THEME	DESCRIPTION	EXAMPLES
Service level implications	misuse of service	Implications for 999 service and unnecessary paramedics call out (Public and other healthcare providers misusing the Ambulance service)	*"The general public will eventually find out and will call 999 just to get a test."*<br><br>*"People will call 999 just to be tested. Also 111 & GPs will send us just to test. We should only be responding if symptoms suggest it's a 999 emergency"*<br><br>*"distracting from the primary cause for 999 calls"*
Service level implications	workload	Concerns regarding additional workload due to SARS-COV-2 testing	*"something else to do"*<br><br>*"additional call outs from patients and other healthcare organisations as a means of rapid testing"*<br><br>*"the wider ambulance service resource demand"*
Service level implications	manage public perception	Comments on managing public opinion or perception of the use of COVID testing in ambulances	*"Patients have been omitting to tell crews about COVID symptoms as they feel they won't get an ambulance if they tell the truth"*<br><br>*"people think we do it anyway"*
Impact on patient care	consequences to care	Impact on the system and on patient care (e.g. use of PPE)	*"False positives/negatives giving false sense of security / resulting in incorrect decisions being made based on that information"*<br><br>*"Culture of wearing PPE would relax"*
Impact on patient care	use case to inform clinical decisions	Concerns around how/who/when/why to test, and effect or impact of the result on decision making or conveyance	*"Does the result really change the outcome especially if leaving someone at home?"*<br><br>*"Why do we need to test? We treat for what we see. If someone is very ill they go to hospital, if they aren't they could stay at home and be referred on. A COVID diagnosis doesn't make a difference"*
Impact on patient care	acceptance	Test acceptance and 'trust' on results	*"Patients have been omitting to tell crews about COVID symptoms as they feel they won't get an ambulance if they tell the truth"*

Impact on patient care	time implications	Delay to service or conveyance due to testing or time to results	*“ensuring it does not delay transfer, especially in time critical patients”*<br><br>*“delay will increase the time the patient will be with the driver and thus may affect the Key performance Indicators”*<br><br>*“increased job cycle times”*<br><br>*“delaying treatment or transport due to waiting for results”*<br><br>*“delays on scene”*<br><br>*“Some people wear level 3 PPE for any COVID +ve patient, meaning if they tested +ve they would then delay things further by going to change PPE”*
Practical use of the test	test feasibility, correct use, and integration with ambulance setting	Usability issues, additional skillset required, appropriate staff training for use of the test and integration with ambulance setting. Storage, portability and stocking of the tests in the ambulance and a paramedic kit	*“How it will be carried - it needs to be introduced into standard response bags that are taken to every patient, similar to our blood sugar testing kits. No one will want to go back to the ambulance to collect a large, bulky box to do it”*<br><br>*“kits won’t be stored correctly due to fluctuating temperatures of a vehicle outside in all weathers”*<br><br>*“keeping in a sterile place”*<br><br>*“Quantity & Supply in order to test volume of patients in a shift (~5-9 per 12 hour shift). Availability of tests for relatives”*<br><br>*“a naso swab could be difficult in a moving vehicle, test would need to be carried out prior to transport”*
Practical use of the test	integration into pathways	Test accuracy and time to results	*“rely on the test having very high specificity and less important but high sensitivity”*<br><br>*“That it is not accurate enough but hospitals will begin to rely on it and not follow up test“*<br><br>*“If it is not specific enough it could lead to clinicians making inaccurate decisions about patient care”*
Safety	risk exposure for staff members	Concerns and comments on risk of exposure to staff	*“Increased risk to staff carrying out [the] test”*<br><br>*“chance of using AGP's then we could use a L3 mask instead”*<br><br>*“That we will increase our on scene time with a positive patient”*
Safety	Staff reassurance	Staff reassurances	*“would assist in reassurance for crews”*

<u>Category 1 - Service level implications</u>

Misuse of service

The major concern from respondents for the roll out of an ambulance based rapid SARS-CoV-2 test was the misuse of the service by the public, appearing in almost half of the responses – *"The general public will eventually find out and will call 999 just to get a test." [IC7]* This concern also expanded into other service providers too – *"People will call 999 just to be tested. Also 111 & GPs will send us just to test. We should only be responding if symptoms suggest it's a 999 emergency" [IC74]*. Many felt that offering SARS-COV-2 testing would result in increased call volume and a resulting increase in call-outs to patients in a non-critical situation *"distracting from the primary cause for 999 calls" [IC158].* These concerns were commonly cited with fear of increased workload for ambulance service staff.

Workload

The potential for additional work to be added in the form of processing and performing tests for SARS-COV-2 prompted some strong, negative responses commonly paired with concerns involving the misuse of the ambulance service. Fundamentally, some service staff considered the SARS-COV-2 test *"something else to do" [IC70]* in an otherwise busy and time critical role. Concerns were raised about *"additional call outs from patients and other healthcare organisations as a means of rapid testing" [IC81]* leading to an impact on the efficiency *and "the wider ambulance service resource demand" [IC55]*.

Public expectation

Responses reflected upon previous experience, with cases of the public not declaring disease or symptoms to avoid incorrect perceived stigma or reduced care – *"Patients have been omitting to tell crews about COVID symptoms as they feel they won't get an ambulance if they tell the truth" [IC42]*. Further concerns stemmed from the overarching belief that the general public may use the ambulance service as a more convenient way to get a SARS-COV-2 test – *"Do not advertise to the public" [IC52], "If testing were to be carried out by ambulances, attempts should be made to keep this away from public knowledge" [IC97]*. Other respondents suggested that the general public already believes that the ambulance services provides routine SARS-COV-2 testing *"people think we do it anyway" [IC178]*, a concern that has already been noted as an issue for some ambulance trusts in previous waves [20].

<u>Category 2 - Impact on patient care</u>

Consequences to care

Confidence in rapid SARS-COV-2 tests appeared to be low with concerns over increased harm for patients and staff associated with erroneous results *"False positives/negatives giving false sense of security / resulting in incorrect decisions being made based on that information" [IC91].* Additional concerns were raised regarding staff adherence to PPE requirements - *"Culture of wearing PPE would relax" [IC108]*.

Informing clinical decisions

Overall, the added value of testing was questioned. Comments on who, when, where or why to test were captured, including - *"Does the result really change the outcome especially if leaving someone at home?" [IC28], "Why do we need to test? We treat for what we see. If someone is very ill they go to hospital, if they aren't they could stay at home and be referred on. A COVID diagnosis doesn't make a difference" [IC94].* Multiple responses inferred that testing would not change decision making, referring to the needs of the patient above or beyond their SARS-COV-2 status – *"Value in most patients limited, other clinical indicators rule COVID in or out, and if they are unable to cope at home they have to be conveyed" [IC145]*.

Acceptance

Few respondents noted concerns with acceptance of testing or acceptability of test results, but did reflect serious implications in the potential rollout of the test, especially regarding informed consent and other ethical considerations when weighed against the inherent risk of a pandemic situation. Representative comments included *"The public refusing to take it" [IC42]* and *"comfort for patients" [IC64]* highlighting concerns over the public reaction to testing.

Time implications

While frequently noted alongside concerns surrounding 'misuse of service' and 'consequences to care', comments directly regarding delays to conveyance as a result of incorporating a SARS-COV-2 test or other knock-on delays to the service were significant. Some responses followed a patient-centric reasoning - *"ensuring it does not delay transfer, especially in time critical patients" [IC14]*, while other comments were concerned about the implications such a delay would have on the ambulance service as a whole – *"delay will increase the time the patient will be with the driver and thus may affect the Key performance Indicator (KPI)" [IC44]* and *"increased job cycle times" [IC162]*. Time to result was a key concern amongst paramedics. This was reflected in the open-response answers *as "delaying treatment or transport due to waiting for results" [IC122], "delays on scene" [IC113]* and *"delaying transport while awaiting results" [IC83]*. Additionally, the use of PPE when working with COVID-19 positive patients was noted as having time implications *"Some people wear level 3 RPE for any covid +ve patient, meaning if they tested +ve they would then delay things further by going to change PPE" [IC161]* implying additional time implications beyond the process of administering the test itself and awaiting results although current guidance considers the use of PPE mandatory in any confirmed or suspected COVID-19 case [21].

<u>Category 3 - Practical use of the test</u>

Test feasibility, correct use, and integration with ambulance setting

Non-accuracy and time-to-result related test characteristics and associated properties such as usability were cited, along with additional direct implications regarding staff training. Size, storage and physical characteristics were commonly referenced in responses - *"How it will be carried - it needs to be introduced into standard response bags that are taken to every patient, similar to our blood sugar testing kits. No one will want to go back to the ambulance to collect*

a large, bulky box to do it" [IC34]. Further elaboration regarding the limitations posed by the settings within an ambulance were raised – *"kits won't be stored correctly due to fluctuating temperatures of a vehicle outside in all weathers" [IC36]* and *"keeping in a sterile place" [IC40].* Issues regarding physical constraints of an ambulance were also invoked though responses *"Quantity & Supply in order to test volume of patients in a shift (~5-9 per 12 hour shift). Availability of tests for relatives" [IC3]* and the ability of users to actually perform the test in such conditions – *"a naso swab could be difficult in a moving vehicle, test would need to be carried out prior to transport" [IC54].*

Integration into pathways

Test characteristics such as optimum test accuracy and time to results in order to inform decision making were quoted in a larger number of responses, frequently alongside comments on 'consequences to care' and 'use case to inform clinical decisions'. Aspects of test accuracy were mentioned, either as requirements for a test to be worthwhile - *"rely on the test having very high specificity and less important but high sensitivity" [IC13]*, or as a concern of poor accuracy leading to inappropriate clinical decision making – *"That it is not accurate enough but hospitals will begin to rely on it and not follow up test" [IC101], "If it is not specific enough it could lead to clinicians making inaccurate decisions about patient care" [IC107].*

Category 4 - Safety

Risk exposure for staff members

Fear of additional harm as a result of testing included not only breaches in personal protective equipment (PPE) as a result of actually conducting the test *"Increased risk to staff carrying out [the] test" [IC55], "chance of using AGP's then we could use a L3 mask instead" [IC129]* and additionally the increased time required during call outs whilst spent conducting the test *"That we will increase our on scene time with a positive patient" [IC36]*.

Staff reassurance

The issue of patient and staff reassurance was covered in a small number of responses - *"would assist in reassurance for crews" [IC30]*.

Follow up Survey 2 - Optimal test characteristics for prioritised use cases

Optimal test characteristics for identified clinical scenarios

Respondents were positive about the most accurate hypothetical test (greater than 95% sensitivity and specificity) for use as a decision aid for referral or for triaging of patients prior to arrival at secondary care facilities, but were critical of any test regardless of accuracy being utilised to inform the use of PPE for ambulance staff [Figure 3].

Triaging of patients prior to arrival at a secondary care facility was the use case with the most positive responses based on the hypothetical tests presented, with both tests 'over 95% accurate' and tests 'over 80% accurate' receiving an average rating of 'Very useful'.

The free-text portion of the follow-up survey was frequently used by respondents to support their answers. The main comments included issues with current ambulance staff compliance with PPE requirements and worry that a negative test may lead to lower compliance with PPE requirements. Some noted that rather than relying on a COVID-19 test, ambulance staff use other clinical indicators to influence decision making, particularly when determining if a patient should be conveyed or kept at home based upon standard indicators of illness severity such as pulse and oxygen saturations.

Discussion

Our results suggest that PoC testing for SARS-CoV-2 within ambulances could add value in particular clinical scenarios. However only an accurate, rapid test would provide the information required to inform clinical decision making. Confidence in the feasibility of rolling out such a test appears limited, an issue which has also been highlighted in other clinical settings [11]. Finger prick blood tests were preferred over other methods, perhaps supported by respondent's prior experience with blood-glucose tests as highlighted in initial scoping questions on prior experience with PoC testing. Small differences in the operation and use of similar PoC tests mean that training and competency requirements would still be necessary and bring with them additional considerations [22].

Generally, the most popular use cases for testing focussed on patient triage, however, the variation in decision making regarding conveyance to hospital is significant between individual ambulance trusts, and test deployment and response is likely to also vary [23]. Greater evidence generation into situational use of SARS-COV-2 diagnostics may reveal patient sub-groups wherein a rapid test influencing the decision making and conveyance of the patient may improve care without substantially impacting the workload and effectiveness of the ambulance service, but significant concerns raised by ambulance service staff would need to be addressed to ensure adherence.

Pragmatically, the TTR of lateral flow devices for SARS-CoV-2 are typically in the range of 15-30 minutes, fitting with the desired characteristics for paramedics as well as national guidance on reducing hospital admission delays [24]. However, the accuracy they achieve in practice is less than the sensitivity and specificity preferred by respondents to facilitate decision making. The gain in accuracy from typical molecular assays may not have a TTR within the 30 minute window desired by the majority of the respondents. However some molecular assays are available with faster TTR which could be evaluated in this setting [25, 26]. An acceptable TTR alone may not alleviate the main concerns raised by ambulance service staff around misuse of the service, additional training requirements and capacity.

Misuse of service leading to increased workload is clearly an important concern, although there is no evidence yet that members of the public have called emergency services to specifically obtain a test. The overwhelming volume of responses with these concerns, from geographically diverse regions suggests that these views are shared across many ambulance trusts around the UK. This may have been a reflection of the pandemic situation during the

For peer review only

conducting of the survey, where ambulance services were experiencing particularly high handover times and some Accident and Emergency departments operating at capacity [27]. It's possible that issues and concerns regarding misuse of the service for the sole purpose of obtaining SARS-CoV-2 tests has been mitigated by the subsequent (5 April 2021) UK government's announcement to make rapid testing available to everyone in England twice weekly [28].

Overall, the results indicate that ambulance staff are prepared to conduct rapid SARS-CoV-2 testing only if they feel it would add value in making early clinical care decisions. The test characteristics required to achieve this may not match currently available PoC tests. Additionally, there were multiple concerns including the impact of additional testing on speed of care delivery and overall service performance as well as the practical constraints of on-board testing and accuracy requirements. Further research is required to further characterise and address these concerns before PoC testing for SARS-CoV-2 could be widely adopted into the ambulance service.

Limitations

Limited piloting of the surveys was performed due to the short timespan available to develop, disseminate, collect and analyse results, whilst the short interval of data capture (03/12/2020 – 11/01/2021) limited the reach and coverage of the survey. Due to the nature of dissemination, there was wide regional disparity in respondents, with the majority of participants located in the East of England and Greater London, where ambulance waiting times and emergency department capacities were stretched during the period of data collection. The collection of data from all members of the ambulance service, while useful at the time of collection, may mean that applicability of some responses to a first-line use cases in ambulances may not be coming from first-hand experience in that setting. The majority of respondents were frontline ambulance staff and paramedics and thus were largely representative of the intended population.

Due to the online interface and open availability, the role and positions of respondents and their understanding of test performance descriptions (i.e. sensitivity and specificity) could not be verified. Descriptions and summaries of these words and phrases were included in the survey to ensure understanding. It was evident that some Trusts had been trialling SARS-CoV-2 PoC testing, while others has refrained, meaning that prior experience with such tests was very variable amongst respondents. The dissemination of the survey primarily through ambulance trust R&D teams and direct contact with respondents may mitigate concerns over false data to an extent.

None of the questions in the surveys were compulsory and most answers provided some free text capacity, those with stronger positive or negative opinions on the subject matter may have been more driven to provide their opinions, introducing a degree of selection bias in participants.

For peer review only

List of abbreviations:

CONDOR - The COVID-19 National DiagnOstic Research and Evaluation Platform

IVD - In-Vitro Diagnostics

LFD - Lateral flow device

NIHR - National Institute for Health Research

PoC – Point of care

PPE - Personal Protective Equipment

R&D - Research and Development

TTR – Time to results

Declarations:

Ethics approval statement:

This work was registered as a service evaluation in Newcastle upon Tyne Hospitals NHS Foundation Trust (project ID: 10641) and granted Caldicott approval (ID: 7867).

Consent for publication:

Consent was obtained from participants after they read the information on the cover page of the survey and agreed to participate by clicking through to the survey questions on the following pages.

Data sharing statement:

Anonymised data used in this publication is available from the corresponding author upon reasonable request

Competing interests:

The authors declare no conflict of interest pertaining to this body of work.

Funding:

This study was part of the CONDOR platform (condor-platform.org), which is funded by the UKRI, Asthma UK and the British Lung Foundation [COV0051]. KG, AW, TH, DAP and AJA are supported by the National Institute for Health Research (NIHR) Newcastle In Vitro Diagnostics Co-operative (http://www.newcastle.mic.nihr.ac.uk/) [MIC-2016-014]. MaM is supported by the NIHR London In Vitro Diagnostics Co-operative

For peer review only

(https://london.ivd.nihr.ac.uk/) [MIC-2016-008]. BS is part-funded as an Associate Director of the NIHR Leeds In Vitro Diagnostics Co-operative (https://www.leedsmic.nihr.ac.uk/) [MIC-2016-015]. The funders had no role in study design, data collection and analysis, decision to publish, or preparation of the manuscript.

Contributorship statement:

AJA and JM conceived the study and design. KG, AW, TH and MAM contributed to the study design. KG developed the surveys. AJA and MAM contributed to survey development. MAM and JM piloted the surveys. JM, KG and AJA disseminated the survey. KG analysed quantitative data. KG, AJA and MAM analysed free-text responses and performed thematic analysis of qualitative responses. TH completed checklist of qualitative reporting. KG and AJA produced the figures and tables. KG and AJA wrote initial manuscript drafts. BS, LS, CP and KD revised and edited the manuscript. All authors reviewed and agreed with the results and conclusions of this manuscript.

Acknowledgements:

The authors would like to thank Graham McClelland for early discussion and scoping for this project, as well as all of the participants who contributed their time and expertise in completing the surveys. The authors are grateful for the input and feedback provided by the MIC Insight Panel and other PPIE members. The authors also acknowledge the support and contributions of the NIHR and CONDOR steering group. Additional members of the CONDOR Steering group include: Dr Emily Adams, Professor Richard Body, Dr Julian Braybrook, Professor Peter Buckle, Professor Paul Dark, Dr Kerrie Davis, Dr Eloïse Cook, Professor Adam Gordon, Mrs Anna Halstead, Dr Charlotte Harden, Dr Colette Inkson, Professor Gail Hayward, Ms Naoko Jones, Professor Dan Lasserson, Dr Joseph Lee, Professor Andrew Lewington, Mrs Mary Logan, Dr Massimo Micocci, Dr Brian Nicholson, Professor Rafael Perera-Salazar, Mr Graham Prestwich, Dr D. Ashley Price, Dr Charles Reynard, Dr Beverley Riley, Professor John Simpson, Mrs Valerie Tate, Dr Philip Turner, Professor Mark Wilcox and Dr Melody Zhifang.

The views expressed in this manuscript are those of the authors and not necessarily those of the NIHR or the Department of Health and Social Care.

References

1. Dinnes, J., et al., *Rapid, point-of-care antigen and molecular-based tests for diagnosis of SARS-CoV-2 infection.* Cochrane Database Syst Rev, 2020. **8**: p. CD013705.
2. FindDX. *FindDX SARS-CoV-2 diagnostic pipeline*. 2021 [cited 2021; Available from: https://www.finddx.org/covid-19/pipeline/.
3. Bossuyt, P.M., et al., *Comparative accuracy: assessing new tests against existing diagnostic pathways.* BMJ, 2006. **332**(7549): p. 1089-92.
4. Ferrante di Ruffano, L., et al., *Assessing the value of diagnostic tests: a framework for designing and evaluating trials.* BMJ, 2012. **344**: p. e686.

For peer review only

5. Garcia-Finana, M., et al., *Performance of the Innova SARS-CoV-2 antigen rapid lateral flow test in the Liverpool asymptomatic testing pilot: population based cohort study.* BMJ, 2021. **374**: p. n1637.
6. Joint PHE Porton Down & University of Oxford SARS-CoV-2 test development and validation cell, *Rapid evalutaion of Lateral Flow Viral Antigen detection devices (LFDs) for mass community testing*. 2021: Oxford University.
7. University of Liverpool, *Innova Lateral Flow SARS-CoV-2 Antigen test accuracy in Liverpool Pilot: preliminary data.* 2020.
8. Medicines & Healthcare products Regulatory Agency, *Target Product Profile: antibody tests to help determine if people have recent infection to SARS-CoV-2: Version 2*. 2021.
9. Graziadio, S., et al., *How to Ease the Pain of Taking a Diagnostic Point of Care Test to the Market: A Framework for Evidence Development.* Micromachines (Basel), 2020. **11**(3).
10. CONDOR steering group. *CONDOR: COVID-19 National DiagnOstic Research and Evaluation Platform*. 2021; Available from: https://www.condor-platform.org/.
11. Graziadio, S., et al., *Unmet clinical needs for COVID-19 tests in UK health and social care settings.* PLoS One, 2020. **15**(11): p. e0242125.
12. Nicholson, B.D., et al., *Rapid community point-of-care testing for COVID-19 (RAPTOR-C19): protocol for a platform diagnostic study.* Diagn Progn Res, 2021. **5**(1): p. 4.
13. Burges Watson, D.L., et al., *Evidence from the scene: paramedic perspectives on involvement in out-of-hospital research.* Ann Emerg Med, 2012. **60**(5): p. 641-50.
14. SurveyMonkey Inc.; Available from: www.surveymonkey.com.
15. Ritchie H, M.E., et al. *Coronavirus Pandemic (COVID-19)*. 2020; Available from: https://ourworldindata.org/coronavirus/country/united-kingdom#how-many-tests-are-performed-each-day.
16. Parliament, U., *COVID-19: NHS Bed Capacity*. 2021.
17. Eysenbach, G., *Improving the quality of Web surveys: the Checklist for Reporting Results of Internet E-Surveys (CHERRIES).* J Med Internet Res, 2004. **6**(3): p. e34.
18. O'Brien, B.C., et al., *Standards for reporting qualitative research: a synthesis of recommendations.* Acad Med, 2014. **89**(9): p. 1245-51.
19. Ritchie, J.L., J; Nicholls, C.M; Ormston, R;, *Qualitative research practice: A guide for social science students and researchers*. 2nd Edition ed. 2013: SAGE Publishing.
20. Association of ambulance chief executives.; Available from: https://aace.org.uk/wp-content/uploads/2020/10/Integrated-Urgent-and-Emergency-Care-Covid-19-Case-Study-240920.pdf.
21. Public Health England. 29/01/2021]; Available from: https://www.gov.uk/government/publications/covid-19-guidance-for-ambulance-trusts/covid-19-guidance-for-ambulance-trusts#personal-protective-equipment.
22. Blanchard, I.E., et al., *Community paramedic point of care testing: validity and usability of two commercially available devices.* BMC Emerg Med, 2019. **19**(1): p. 30.
23. O'Cathain, A., et al., in *Understanding variation in ambulance service non-conveyance rates: a mixed methods study*. 2018: Southampton (UK).
24. Lord Carter, *Operational productivity and performance in English NHS Ambulance Trusts: Unwarranted variations*. 2018.
25. Dinnes, J., et al., *Rapid, point-of-care antigen and molecular-based tests for diagnosis of SARS-CoV-2 infection.* Cochrane Database Syst Rev, 2021. **3**: p. CD013705.
26. Schildgen, V., et al., *Limits and Opportunities of SARS-CoV-2 Antigen Rapid Tests: An Experienced-Based Perspective.* Pathogens, 2021. **10**(1).
27. England, N., *Urgent and Emergency care daily situation reports 2020-21*, N. England, Editor. 2021.

For peer review only

28. UK government. *Twice weekly rapid testing to be available to everyone in England*. 2021; Available from: https://www.gov.uk/government/news/twice-weekly-rapid-testing-to-be-available-to-everyone-in-england.

Figure 1: Summary of use cases for rapid SARS-COV-2 testing in ambulances

170 respondents stated their preferred use cases for a rapid SARS-COV-2 test in the ambulance service as displayed in this stacked chart. The length of the bar represents the number of respondents providing that use case, with the colours representing the categories of ambulance service staff. A call-out text box displays the main quotes from respondents that noted 'other' as their preferred use case.

Figure 2: Summary scores for most important test characteristics for SARS-COV-2 testing in an ambulance setting

Summary scores for the Likert ranking of test characteristics for a rapid SARS-COV-2 test in the ambulance service are displayed in this chart. Likert scores ranged from -2 [Not important] to +2 [Most important]. Mean score and standard deviation of the results are displayed in the figure.

Figure 3: Summary scores for perceived usefulness of hypothetical SARS-COV-2 tests for top use cases identified though survey.

Summary scores for the Likert ranking of use cases and accuracy statistics for a rapid SARS-COV-2 test in the ambulance service are displayed in this chart. Likert scores ranged from -2 [Not important] to +2 [Most important]. The length of the bars indicates the mean Likert score, while the colour represents the three hypothetical tests referred to in the figure key.

For peer review only

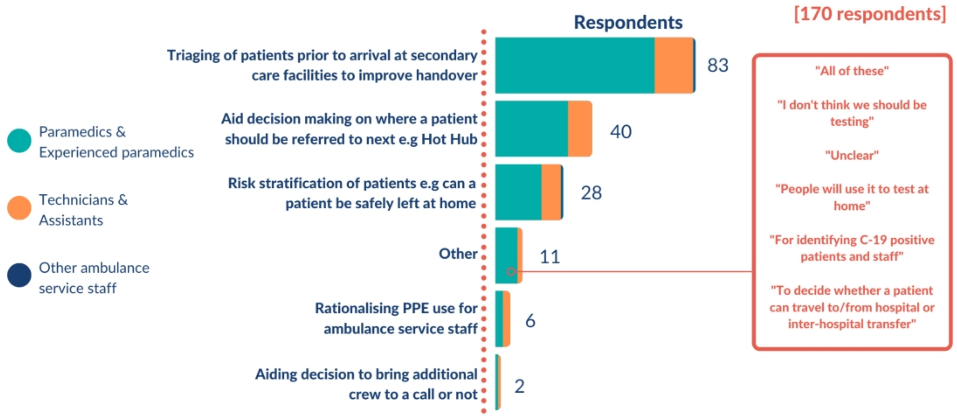

861x484mm (59 x 59 DPI)

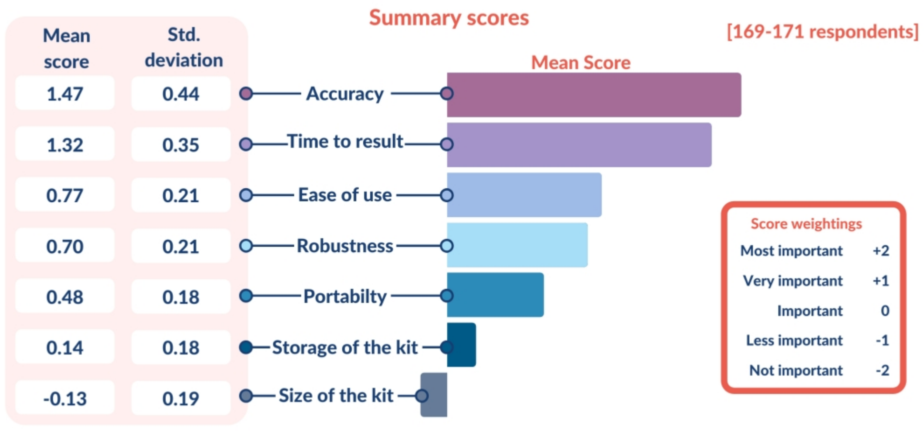

861x484mm (59 x 59 DPI)

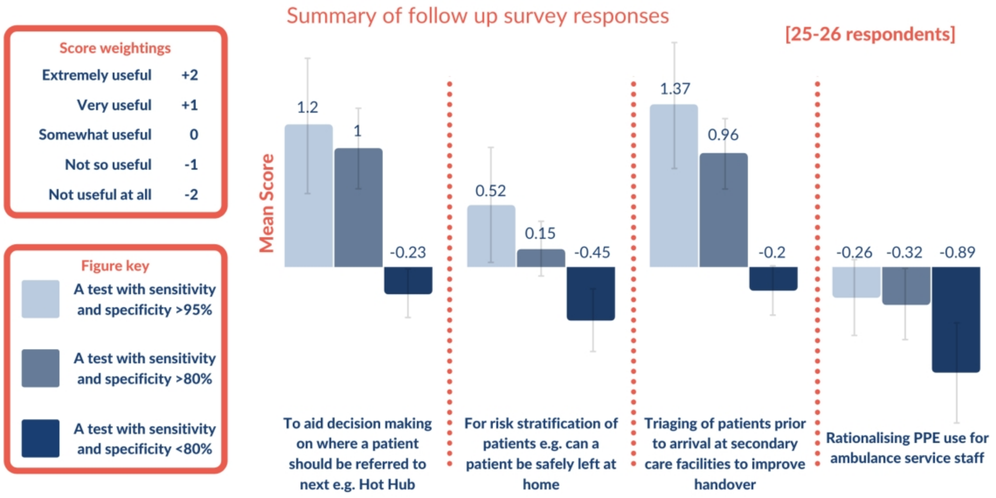

861x484mm (59 x 59 DPI)

Supplementary Material 1: Survey 1 questions

Page 1: Landing page

COVID-19 rapid testing within the Ambulance Service

Dear Participant,

The aim of this survey is to understand the potential role and perceived value of rapid testing within the ambulance service.

You have been invited to participate because you are a health or social care professional in UK.

The survey should take no more than 3 minutes to complete.

No personal or sensitive data is required, and your response is anonymous and will be kept confidential.

Results will be used to inform future research in this area.

If you have any questions or would like to get in touch please do not hesitate to contact: nihr.newcastle.mic@ncl.ac.uk.

By continuing to the next page you will be consenting to participate in this survey.

Thank you for taking part.

Page 2: Participant Information

1. **Which region of the UK do you work in?**

Scotland

Northern Ireland

Wales

North West England

North East England

Yorkshire & The Humber

West Midlands

East Midlands

South West

South East

East of England

Greater London

2. **What is your current job role?**

Ambulance Service Team – Ambulance care assistant and Patient Transport Service (PTS) driver

Ambulance Service Team – Call handler/emergency medical dispatcher

Ambulance Service Team – Emergency care assistant

Ambulance Service Team – Emergency medical technician

Ambulance Service Team - Paramedic

Ambulance Service Team – Experience paramedic

peer review only

Ambulance Service Team – Patient Transport Service (PTS) call handler

Ambulance Service Team – Integrated urgent care/NHS 111

3. **Please use this space to add any other information regarding your role, if applicable (eg. specialism, additional expertise, area of interest)**

    [Free Text]

Page 3: COVID-19 rapid testing within the Ambulance Service

4. **Do you already conduct any tests for respiratory infection in a pre-hospital setting? If yes, please describe the test(s) below:**

    [Free text]

5. **How feasible do you think it would be to test patients for COVID-19 before transfer to hospital?**

    Very high

    High

    Neutral

    Low

    Very Low

    a. **Please add any additional comments on feasibility here:**

    [Free text]

6. **What would be the optimal or ideal sampling technique for a COVID-19 test in an ambulance setting?**

    Nasopharyngeal swab

    Finger prick blood test

    Sputum sample

    Saliva sample

    Other (please specify)

    [Free text]

7. **How helpful do you feel the deployment of rapid COVID-19 testing in an ambulance setting would be?**

    Very helpful

    Quite helpful

    Neither helpful nor unhelpful

    Quite unhelpful

    Very unhelpful

8. **What do you think the main use case for rapid COVID-19 testing in ambulances would be?**

For peer review only

Triaging of patients prior to arrival at secondary care facilities to improve handover and flow

Risk stratification of patients e.g. can a patient be safely left at home

Aid decision making on where the patient should be referred to next e.g. Hot-Hub

Rationalising PPE use for ambulance service staff

Aiding decision to bring additional crew to a call or not

Other (please specify)

[Free Text]

9. What do you think the most important characteristics of a test on board ambulances would be?

Time to result – Most important – very important – important – less important – not important

Accuracy – Most important – very important – important – less important – not important

Ease of use – Most important – very important – important – less important – not important

Robustness – Most important – very important – important – less important – not important

Size of the kit – Most important – very important – important – less important – not important

Storage of the kit – Most important – very important – important – less important – not important

Portability of the kit – Most important – very important – important – less important – not important

Other (please specify) – Most important – very important – important – less important – not important

10. What would be the maximum acceptable time to results (TTR) for an ambulance-based COVID test?

[Slider scale 'Time to results in minutes', Minimum value = 0, Maximum value = 45]

11. What is the minimum sensitivity that a test in ambulances should have?

>60% (at least 6/10 people with COVID will get a positive result)

>70% (at least 7/10 people with COVID will get a positive result)

>80% (at least 8/10 people with COVID will get a positive result)

>90% (at least 9/10 people with COVID will get a positive result)

12. What is the minimum specificity that a test in ambulances should have?

>60% (at least 6/10 people without COVID will get a negative result)

>70% (at least 7/10 people without COVID will get a negative result)

>80% (at least 8/10 people without COVID will get a negative result)

>90% (at least 9/10 people without COVID will get a negative result)

13. What do you think the main concerns of adding a test on board an ambulance would be?

[Free Text]

For peer review only

14. Is there anything else you feel is important to tell us about testing in ambulances?

[Free Text]

15. If you would be willing to speak to us further about COVID-19 testing within the ambulance service, please provide details below

[Name]

[Preferred Email Address]

For peer review only

Supplementary Material 2: Survey 2 questions

Page 1: Landing page

Use cases and diagnostic profiles for COVID-19 rapid testing within the Ambulance Service

Dear Participant,

The aim of this survey is to further understand the potential role for COVID-19 diagnostic tests in different scenarios relevant to the ambulance service.

You have been invited to participate because you are a health or social care professional in UK and have previously provided contact details for us to speak to you further about COVID-19 testing in an ambulance setting.

The follow up survey should take no more than 3 minutes to complete.

No personal or sensitive data is required, and your response is anonymous and will be kept confidential.

Results will be used to inform future research in this area.

If you have any questions or would like to get in touch please do not hesitate to contact: nihr.newcastle.mic@ncl.ac.uk.

By continuing to the next page you will be consenting to participate in this survey.

Thank you for taking part.

Page 2: Use cases and diagnostic profiles for COVID-19 rapid testing within the Ambulance Service

The results of our initial survey highlighted a number of potential use cases that participants felt may be important. In this follow up series of questions we would like to better understand the qualities and characteristics of COVID-19 tests that you feel are necessary for each use case.

Please rate how useful you feel each type of test would be for each of the use cases from 'Extremely useful' to 'Not at all useful'

Sensitivity - The ability of a test to correctly identify a patient with the disease.
[The test is positive when the patient has COVID-19]

Specificity - The ability of a test to correctly identify a patient who does not have the disease.
[The test is negative when the patient does not have COVID-19]

1. **A test with greater than 95% sensitivity and specificity, less than 15 minutes time to results**
    a. Aid decision making on where the patient should be referred to next e.g. Hot-Hub

        Extremely useful – Very useful – Somewhat useful – Not so useful – Not at all useful

    b. Risk stratification of patients e.g. can a patient be safely left at home

        Extremely useful – Very useful – Somewhat useful – Not so useful – Not at all useful

    c. Triaging of patients prior to arrival at secondary care facilities to improve handover and flow

        Extremely useful – Very useful – Somewhat useful – Not so useful – Not at all useful

    d. Rationalising PPE use for ambulance service staff

        Extremely useful – Very useful – Somewhat useful – Not so useful – Not at all useful

- Please provide any additional comments or reasoning here:

  [Free Text]

2. **A test with greater than 80% sensitivity and specificity, less than 15 minutes time to results**
    a. Aid decision making on where the patient should be referred to next e.g. Hot-Hub

Extremely useful – Very useful – Somewhat useful – Not so useful – Not at all useful

b. Risk stratification of patients e.g. can a patient be safely left at home

Extremely useful – Very useful – Somewhat useful – Not so useful – Not at all useful

c. Triaging of patients prior to arrival at secondary care facilities to improve handover and flow

Extremely useful – Very useful – Somewhat useful – Not so useful – Not at all useful

d. Rationalising PPE use for ambulance service staff

Extremely useful – Very useful – Somewhat useful – Not so useful – Not at all useful

- Please provide any additional comments or reasoning here:

[Free Text]

3. A test with less than 80% sensitivity and specificity, less than 15 minutes time to results

a. Aid decision making on where the patient should be referred to next e.g. Hot-Hub

Extremely useful – Very useful – Somewhat useful – Not so useful – Not at all useful

b. Risk stratification of patients e.g. can a patient be safely left at home

Extremely useful – Very useful – Somewhat useful – Not so useful – Not at all useful

c. Triaging of patients prior to arrival at secondary care facilities to improve handover and flow

Extremely useful – Very useful – Somewhat useful – Not so useful – Not at all useful

d. Rationalising PPE use for ambulance service staff

Extremely useful – Very useful – Somewhat useful – Not so useful – Not at all useful

- Please provide any additional comments or reasoning here:

[Free Text]

For peer review only

COMPLETION OF THIS CHECKLIST WAS REQUESTED BY REVIEWERS OF THE INITIAL EMJ SUBMISSION

Item Category	Checklist Item	Explanation	Manuscript Response/Description
**Design**	Describe survey design	Describe target population, sample frame. Is the sample a convenience sample? (In "open" surveys this is most likely.)	*The target population was emergency medical staff working in the UK. The survey was 'live' for a set time and was an open survey disseminated by Trust R&D teams and targeted individuals.*
**IRB (Institutional Review Board) approval and informed consent process**	IRB approval	Mention whether the study has been approved by an IRB.	*The project was approved as a service evaluation by NuTH and each participating trust decided internally to disseminate amongst staff.*
	Informed consent	Describe the informed consent process. Where were the participants told the length of time of the survey, which data were stored and where and for how long, who the investigator was, and the purpose of the study?	*The landing page of the survey detailed the purpose of the survey, the estimated length of time of the survey and that sharing personal information was not a required part of the survey. The investigator and contact details were listed on the landing page.*
	Data protection	If any personal information was collected or stored, describe what mechanisms were used to protect unauthorized access.	*All data collected form the survey was downloaded to a password protected computer and stored in a locked room in a Newcastle University building.*
**Development and pre-testing**	Development and testing	State how the survey was developed, including whether the usability and technical functionality of the electronic questionnaire had been tested before fielding the questionnaire.	*The survey was piloted internally within the research group and externally with choice experts. Feedback from the pilot was used to refine and edit the final survey. The results from piloting the survey were not included in the final manuscript.*
**Recruitment process and description of the sample having access to the questionnaire**	Open survey versus closed survey	An "open survey" is a survey open for each visitor of a site, while a closed survey is only open to a sample which the investigator knows (password-protected survey).	*The survey was an open survey in that access to the survey could be made by anyone with the survey-specific link. The link was shared directly with respondents and disseminated by relevant persons.*
	Contact mode	Indicate whether or not the initial contact with the potential participants was made on the Internet. (Investigators may also send out questionnaires by mail and allow for Web-based data entry.)	*The main mode of contact was via email. The link and supporting information was sent directly to participants email addresses.*
	Advertising the survey	How/where was the survey announced or advertised? Some examples are offline media (newspapers), or online (mailing lists – If yes, which ones?) or banner ads (Where were these banner ads posted and what did they look like?). It is important to know the wording of the announcement as it will heavily influence who chooses to participate. Ideally the survey announcement should be published as an appendix.	*The survey was not advertised.*
**Survey administration**	Web/E-mail	State the type of e-survey (eg, one posted on a Web site, or one sent out	*The survey was web-based. There was no manual copying of the*

For peer review only

COMPLETION OF THIS CHECKLIST WAS REQUESTED BY REVIEWERS OF THE INITIAL EMJ SUBMISSION

		through e-mail). If it is an e-mail survey, were the responses entered manually into a database, or was there an automatic method for capturing responses?	*responses into a database. Responses were captured automatically.*
	Context	Describe the Web site (for mailing list/newsgroup) in which the survey was posted. What is the Web site about, who is visiting it, what are visitors normally looking for? Discuss to what degree the content of the Web site could pre-select the sample or influence the results. For example, a survey about vaccination on a anti-immunization Web site will have different results from a Web survey conducted on a government Web site	*The survey was not posted to a website. It was disseminated directly via email.*
	Mandatory/voluntary	Was it a mandatory survey to be filled in by every visitor who wanted to enter the Web site, or was it a voluntary survey?	*The survey was voluntary and no answers were mandatory.*
	Incentives	Were any incentives offered (eg, monetary, prizes, or non-monetary incentives such as an offer to provide the survey results)?	*No incentives were offered.*
	Time/Date	In what timeframe were the data collected?	*The data was collected during the COVID-19 pandemic. Survey 1 was launched on 3rd December 2020 and was open to responses until 11th January 2021. Survey 2 was disseminated to interested parties on 8th February 2021 and was open to responses until 22nd February 2021.*
	Randomization of items or questionnaires	To prevent biases items can be randomized or alternated.	*Randomisation was not performed. Questions generally followed logically and would have been difficult to interpret out of order.*
	Adaptive questioning	Use adaptive questioning (certain items, or only conditionally displayed based on responses to other items) to reduce number and complexity of the questions.	*Questions involving an 'other' or 'NA' option would generate a free-text box where respondents could expand on their answer.*
	Number of Items	What was the number of questionnaire items per page? The number of items is an important factor for the completion rate.	*Survey 1 had 3 questions on page 1, and 12 questions on page 2. Survey 2 had 3 questions on a single page.*
	Number of screens (pages)	Over how many pages was the questionnaire distributed? The number of items is an important factor for the completion rate.	*Survey 1 was composed of 4 pages, 2 of which had questions: Landing page, page 1 'demographics', page 2 'COVID-19 testing', Final page 'Survey Complete'. Survey 2 was composed of 3 pages, 1 of which had questions: Landing page, page 1 'follow up questions', Final page 'Survey Complete'.*
		It is technically possible to do	*The survey software noted complete*

For peer review only

COMPLETION OF THIS CHECKLIST WAS REQUESTED BY REVIEWERS OF THE INITIAL EMJ SUBMISSION

	Completeness check	consistency or completeness checks before the questionnaire is submitted. Was this done, and if "yes", how (usually JAVAScript)? An alternative is to check for completeness after the questionnaire has been submitted (and highlight mandatory items). If this has been done, it should be reported. All items should provide a non-response option such as "not applicable" or "rather not say", and selection of one response option should be enforced.	*response to the survey questions or noted at which page the survey was prematurely ended.*
	Review step	State whether respondents were able to review and change their answers (eg, through a Back button or a Review step which displays a summary of the responses and asks the respondents if they are correct).	*Reponses could be changed at any point during the survey by altering answers or using the 'back' and 'forward' buttons to navigate between pages.*
**Response rates**	Unique site visitor	If you provide view rates or participation rates, you need to define how you determined a unique visitor. There are different techniques available, based on IP addresses or cookies or both.	*Each respondent was assigned a unique numerical ID upon starting the survey.*
	View rate (Ratio of unique survey visitors/unique site visitors)	Requires counting unique visitors to the first page of the survey, divided by the number of unique site visitors (not page views!). It is not unusual to have view rates of less than 0.1 % if the survey is voluntary.	*This information would be misleading. The view rate of the surveys was high based on visitors to the first page. This was because the survey was disseminated via email and those that were interested would have clicked on the survey link, while those who were not interested would not have responded to the email, or clicked the link. The reach of the survey is unknown due to the varied dissemination routes. All visitors to the site were linked directly to the first 'landing page' of the survey.*
	Participation rate (Ratio of unique visitors who agreed to participate/unique first survey page visitors)	Count the unique number of people who filled in the first survey page (or agreed to participate, for example by checking a checkbox), divided by visitors who visit the first page of the survey (or the informed consents page, if present). This can also be called "recruitment" rate.	*As above, this value would be misleading as only those potential participants that were interested in completing the survey would visit the weblink supplied in their email. Based on page views, all visitors clicked through the first page of the survey. The reach of the survey is unknown due to method of dissemination.*
	Completion rate (Ratio of users who finished the survey/users who agreed to participate)	The number of people submitting the last questionnaire page, divided by the number of people who agreed to participate (or submitted the first survey page). This is only relevant if there is a separate "informed consent" page or if the survey goes over several pages. This is a measure for attrition. Note that "completion" can involve leaving questionnaire items blank. This	*For purposes of this checklist - One respondent in survey 1 did not complete any answers, all others did. 211/212 = 99.5%.*<br>*Reach of the survey is unknown.*<br><br>*For survey 2, all respondents who started the survey, finished it. 26/26 = 100%*<br>*However, as noted in the above*

For peer review only

COMPLETION OF THIS CHECKLIST WAS REQUESTED BY REVIEWERS OF THE INITIAL EMJ SUBMISSION

		is not a measure for how completely questionnaires were filled in. (If you need a measure for this, use the word "completeness rate".)	*sections, this number is misleading as survey 2 was emailed to 50 participants, with 26 clicking the link to the survey and participating in it. 26/50 = 52% would be a more accurate reflection of the number of participants who agreed to do the second survey and those who actually participated.*
**Preventing multiple entries from the same individual**	Cookies used	Indicate whether cookies were used to assign a unique user identifier to each client computer. If so, mention the page on which the cookie was set and read, and how long the cookie was valid. Were duplicate entries avoided by preventing users access to the survey twice; or were duplicate database entries having the same user ID eliminated before analysis? In the latter case, which entries were kept for analysis (eg, the first entry or the most recent)?	*Each user was assigned a unique numerical ID upon starting the survey. There were no duplicate IDs noted in the results.*<br>*We are not able to rule out the possibility of respondents completing the survey from multiple devices or browsers as anyone with the relevant weblink could access the survey.*
	IP check	Indicate whether the IP address of the client computer was used to identify potential duplicate entries from the same user. If so, mention the period of time for which no two entries from the same IP address were allowed (eg, 24 hours). Were duplicate entries avoided by preventing users with the same IP address access to the survey twice; or were duplicate database entries having the same IP address within a given period of time eliminated before analysis? If the latter, which entries were kept for analysis (eg, the first entry or the most recent)?	*Each user was assigned a unique numerical ID upon starting the survey. There were no duplicate IDs noted in the results.*<br>*We are not able to rule out the possibility of respondents completing the survey from multiple devices or browsers as anyone with the relevant weblink could access the survey.*
	Log file analysis	Indicate whether other techniques to analyze the log file for identification of multiple entries were used. If so, please describe.	*This was not performed.*
	Registration	In "closed" (non-open) surveys, users need to login first and it is easier to prevent duplicate entries from the same user. Describe how this was done. For example, was the survey never displayed a second time once the user had filled it in, or was the username stored together with the survey results and later eliminated? If the latter, which entries were kept for analysis (eg, the first entry or the most recent)?	*Once a user had completed the survey once, that same user would not be able to complete the survey again.*<br>*Users may have been able to circumvent this by changing browsers or using an alternative device.*<br>*Access to the survey was not password protected.*
**Analysis**	Handling of incomplete questionnaires	Were only completed questionnaires analyzed? Were questionnaires which terminated early (where, for example, users did not go through all questionnaire pages) also analyzed?	*One respondent did not complete any answers. This instance was removed from the final analysis.*<br>*All questions were optional, the figures in the manuscript reflect the number of responses received to*

For peer review only - http://bmjopen.bmj.com/site/about/guidelines.xhtml

COMPLETION OF THIS CHECKLIST WAS REQUESTED BY REVIEWERS OF THE INITIAL EMJ SUBMISSION

			each individual question.
	Questionnaires submitted with an atypical timestamp	Some investigators may measure the time people needed to fill in a questionnaire and exclude questionnaires that were submitted too soon. Specify the timeframe that was used as a cut-off point, and describe how this point was determined.	*The survey was relatively short. There was no minimum time limit for responses to be considered valid.*
	Statistical correction	Indicate whether any methods such as weighting of items or propensity scores have been used to adjust for the non-representative sample; if so, please describe the methods.	*Likert scores were used in both surveys with responses weighted as described in the manuscript. Weighting of individual participant's responses above or below others was not performed.*

For peer review only

Standards for Reporting Qualitative Research (SRQR)*

http://www.equator-network.org/reporting-guidelines/srqr/

Title and abstract

	Page/line no(s).
**Title** - Concise description of the nature and topic of the study Identifying the study as qualitative or indicating the approach (e.g., ethnography, grounded theory) or data collection methods (e.g., interview, focus group) is recommended	Page 1/Lines 1-4
**Abstract** - Summary of key elements of the study using the abstract format of the intended publication; typically includes background, purpose, methods, results, and conclusions	Pages 1-2/Lines 21-47

Introduction

**Problem formulation** - Description and significance of the problem/phenomenon studied; review of relevant theory and empirical work; problem statement	Page 3/Lines 52-84
**Purpose or research question** - Purpose of the study and specific objectives or questions	Page 3/Lines 77-84, Page 5, 136-151

Methods

**Qualitative approach and research paradigm** - Qualitative approach (e.g., ethnography, grounded theory, case study, phenomenology, narrative research) and guiding theory if appropriate; identifying the research paradigm (e.g., postpositivist, constructivist/ interpretivist) is also recommended; rationale**	Pages 3-4/Lines 94-115
**Researcher characteristics and reflexivity** - Researchers' characteristics that may influence the research, including personal attributes, qualifications/experience, relationship with participants, assumptions, and/or presuppositions; potential or actual interaction between researchers' characteristics and the research questions, approach, methods, results, and/or transferability	Page 4/ Lines 94-115
**Context** - Setting/site and salient contextual factors; rationale**	Page 3-4/ Lines 94-115, 117-123
**Sampling strategy** - How and why research participants, documents, or events were selected; criteria for deciding when no further sampling was necessary (e.g., sampling saturation); rationale**	Page 4-5/Lines 126-133
**Ethical issues pertaining to human subjects** - Documentation of approval by an appropriate ethics review board and participant consent, or explanation for lack thereof; other confidentiality and data security issues	Page 15 / Lines 449-456
**Data collection methods** - Types of data collected; details of data collection procedures including (as appropriate) start and stop dates of data collection and analysis, iterative process, triangulation of sources/methods, and modification of procedures in response to evolving study findings; rationale**	Pages 4-5, Lines 95-133

For peer review only

For peer review only - http://bmjopen.bmj.com/site/about/guidelines.xhtml

**Data collection instruments and technologies** - Description of instruments (e.g., interview guides, questionnaires) and devices (e.g., audio recorders) used for data collection; if/how the instrument(s) changed over the course of the study	Pages 5-6/Lines 158-174
**Units of study** - Number and relevant characteristics of participants, documents, or events included in the study; level of participation (could be reported in results)	Page 4 & 6 /Lines 103 – 115, 176-195
**Data processing** - Methods for processing data prior to and during analysis, including transcription, data entry, data management and security, verification of data integrity, data coding, and anonymization/de-identification of excerpts	Pages 5-6/Lines 158-174
**Data analysis** - Process by which inferences, themes, etc., were identified and developed, including the researchers involved in data analysis; usually references a specific paradigm or approach; rationale**	Pages 5-6/Lines 158-174
**Techniques to enhance trustworthiness** - Techniques to enhance trustworthiness and credibility of data analysis (e.g., member checking, audit trail, triangulation); rationale**	Page 4 & 6/Lines 96-101, 167-174

Results/findings

**Synthesis and interpretation** - Main findings (e.g., interpretations, inferences, and themes); might include development of a theory or model, or integration with prior research or theory	Pages 6-13 /Lines 175-364
**Links to empirical data** - Evidence (e.g., quotes, field notes, text excerpts, photographs) to substantiate analytic findings	Pages 6-13 /Lines 175-364

Discussion

**Integration with prior work, implications, transferability, and contribution(s) to the field -** Short summary of main findings; explanation of how findings and conclusions connect to, support, elaborate on, or challenge conclusions of earlier scholarship; discussion of scope of application/generalizability; identification of unique contribution(s) to scholarship in a discipline or field	Page 3 & 13-14/Lines 69-84, 365-410
**Limitations** - Trustworthiness and limitations of findings	Pages 14/Lines 411-434

Other

**Conflicts of interest** - Potential sources of influence or perceived influence on study conduct and conclusions; how these were managed	Page 15/ Lines 462-463
**Funding** - Sources of funding and other support; role of funders in data collection, interpretation, and reporting	Page 15/ Lines 464-473

*The authors created the SRQR by searching the literature to identify guidelines, reporting standards, and critical appraisal criteria for qualitative research; reviewing the reference lists of retrieved sources; and contacting experts to gain feedback. The SRQR aims to improve the transparency of all aspects of qualitative research by providing clear standards for reporting qualitative research.

For peer review only

**The rationale should briefly discuss the justification for choosing that theory, approach, method, or technique rather than other options available, the assumptions and limitations implicit in those choices, and how those choices influence study conclusions and transferability. As appropriate, the rationale for several items might be discussed together.

Reference:

O'Brien BC, Harris IB, Beckman TJ, Reed DA, Cook DA. **Standards for reporting qualitative research: a synthesis of recommendations.** *Academic Medicine*, Vol. 89, No. 9 / Sept 2014 DOI: 10.1097/ACM.0000000000000388

For peer review only

For peer review only - http://bmjopen.bmj.com/site/about/guidelines.xhtml

BMJ Open

The perceived feasibility, facilitators and barriers to incorporating point-of-care testing for SARS-CoV-2 into Emergency Medical Services by Ambulance Service staff: A survey-based approach

Journal:	*BMJ Open*
Manuscript ID	bmjopen-2022-064038.R1
Article Type:	Original research
Date Submitted by the Author:	07-Sep-2022
Complete List of Authors:	Green, Kile; Newcastle University, NIHR Newcastle In Vitro Diagnostics Co-operative, Translational and Clinical Research Institute<br>Micocci, Massimo; Imperial College London, NIHR London In Vitro Diagnostics Co-operative<br>Hicks, Timothy; Newcastle Upon Tyne Hospitals NHS Foundation Trust; Newcastle University, NIHR Newcastle In Vitro Diagnostics Co-operative, Translational and Clinical Research Institute<br>Winter, Amanda; Newcastle Upon Tyne Hospitals NHS Foundation Trust; Newcastle University, NIHR Newcastle In Vitro Diagnostics Co-operative, Translational and Clinical Research Institute<br>Martin, Joanne E; Barts and The London NHS Trust, Centre for Genomics and Child Health, Blizard Institute,<br>Shinkins, Bethany; University of Leeds, Academic Unit of Health Economics<br>Shaw, Lisa; Newcastle University, Stroke Research Group, Population Health Sciences,<br>Price, Christopher; Newcastle University, Institute of Neuroscience,<br>Davies, Kerrie; University of Leeds, Healthcare Associated Infections Research Group<br>Allen, A; Newcastle University, NIHR Newcastle In Vitro Diagnostics Co-operative, Translational and Clinical Research Institute; Newcastle Upon Tyne Hospitals NHS Foundation Trust
**Primary Subject Heading**:	Emergency medicine
Secondary Subject Heading:	Infectious diseases, Diagnostics, Qualitative research
Keywords:	COVID-19, QUALITATIVE RESEARCH, Molecular diagnostics < INFECTIOUS DISEASES

SCHOLARONE™ Manuscripts

I, the Submitting Author has the right to grant and does grant on behalf of all authors of the Work (as defined in the below author licence), an exclusive licence and/or a non-exclusive licence for contributions from authors who are: i) UK Crown employees; ii) where BMJ has agreed a CC-BY licence shall apply, and/or iii) in accordance with the terms applicable for US Federal Government officers or employees acting as part of their official duties; on a worldwide, perpetual, irrevocable, royalty-free basis to BMJ Publishing Group Ltd ("BMJ") its licensees and where the relevant Journal is co-owned by BMJ to the co-owners of the Journal, to publish the Work in this journal and any other BMJ products and to exploit all rights, as set out in our licence.

The Submitting Author accepts and understands that any supply made under these terms is made by BMJ to the Submitting Author unless you are acting as an employee on behalf of your employer or a postgraduate student of an affiliated institution which is paying any applicable article publishing charge ("APC") for Open Access articles. Where the Submitting Author wishes to make the Work available on an Open Access basis (and intends to pay the relevant APC), the terms of reuse of such Open Access shall be governed by a Creative Commons licence – details of these licences and which Creative Commons licence will apply to this Work are set out in our licence referred to above.

Other than as permitted in any relevant BMJ Author's Self Archiving Policies, I confirm this Work has not been accepted for publication elsewhere, is not being considered for publication elsewhere and does not duplicate material already published. I confirm all authors consent to publication of this Work and authorise the granting of this licence.

For peer review only

The perceived feasibility, facilitators and barriers to incorporating point-of-care testing for SARS-CoV-2 into Emergency Medical Services by Ambulance Service staff: A survey-based approach

Dr Kile Green*[1], Dr Massimo Micocci[2], Mr Timothy Hicks[1], Ms Amanda Winter[1], Prof Joanne E Martin[3], Dr Bethany Shinkins[4], Dr Lisa Shaw[5], Professor Christopher I. Price[5], Dr Kerrie Davies[6,7], Dr A. Joy Allen[1] on behalf of the CONDOR Steering group

[1:] NIHR Newcastle In Vitro Diagnostics Co-operative, Newcastle-upon-Tyne Hospitals Foundation Trust, Newcastle, United Kingdom.

[2:] NIHR London In Vitro Diagnostics Co-operative, Imperial College London, London, United Kingdom

[3:] Centre for Genomics and Child Health, Blizard Institute, Barts and The London School of Medicine and Dentistry, Queen Mary, University of London, London, United Kingdom.

[4:] Test Evaluation Group, Leeds Institute for Health Sciences, University of Leeds, Leeds, UK

[5:] Stroke Research Group, Population Health Sciences Institute, Newcastle University, UK.

[6:] Healthcare Associated Infections Research Group, University of Leeds and Leeds Teaching Hospitals NHS Trust, Leeds, UK

[7:] NIHR Leeds In Vitro Diagnostics Co-operative, Leeds Teaching Hospitals NHS Trust, Leeds, UK

** Corresponding author [Dr Kile Green, kile.green@newcastle.ac.uk]*

Abstract

Objectives: This body of work aimed to elicit ambulance service staff's perceptions on the barriers and facilitators to adoption, and clinical utility of incorporating rapid SARS-CoV-2 testing during ambulance assessments.

Design: A mixed-methods survey-based project utilising a Framework Analysis Method to organise qualitative data.

Setting: Emergency and non-emergency care ambulatory services in the UK were approached to take part.

Participants: Current, practicing members of the UK ambulance service (paramedics, technicians, assistants and other staff) were included in this body of work.

Results: Survey 1: 226 responses were collected between 3rd December 2020 and 11th January 2021, 179 (79.2%) of which were completed in full. While the majority of respondents indicated that an ambulance-based testing strategy was feasible in concept (143/190, 75.3%), major barriers to adoption were noted. Many open-ended responses cited concerns regarding misuse of the service by the general public and other healthcare services, timing

For peer review only

and conveyance issues, and increased workloads, alongside training and safety concerns. Survey 2: 26 responses were received between 8th February 2021 and 22nd February 2021 to this follow-up survey. Survey 2 revealed conveyance decision-making, and risk stratification to be the most frequently prioritised use cases amongst ambulance service staff. Optimal test characteristics for clinical adoption according to respondents were; accuracy (above 90% sensitivity and specificity), rapidity (<30 minutes time to results) and ease of sample acquisition.

Conclusions: The majority of commercially available lateral flow devices are unlikely to be supported by paramedics as their duty of care requires both rapid and accurate results that can inform clinical decision making in an emergency situation. Further investigation is needed to define acceptable test characteristics and criteria required for ambulance service staff to be confident and supportive of deployment of a SARS-CoV-2 test in an emergency care setting.

Keywords: Ambulance Service, COVID-19, feasibility, point-of-care testing, SARS-CoV-2, survey, thematic analysis.

For peer review only

Strengths and limitations of this study

- Strength – This body of work represents a mixed-methods survey-based approach to determine feasibility of SARS-CoV-2 testing in ambulances, capturing qualitative and quantitative outputs from frontline healthcare workers from all regions of England, and Northern Ireland.
- Strength – The inclusion of open-ended questions allowed for in-depth responses to be captured from respondents, providing additional context and reasoning.
- Limitation – Limited piloting of the surveys was performed due to the short timespan available to develop, disseminate, collect and analyse results, whilst the short interval of data capture (03/12/2020 – 11/01/2021) limited the reach and coverage of the survey.
- Limitation – The low (52%) response rate to the follow-up survey (Survey 2) may have introduced a degree of bias and represents a much more limited dataset than Survey 1.

Background

In response to the COVID-19 pandemic, there was large scale production of rapid, portable clinical tests for SARS-CoV-2 with CE-marking for in vitro diagnostics (CE-IVD) approval [1, 2]. Like any test, their true value hinges on their role within a clinical pathway, the real-world accuracy of the test within that role, and the efficacy of the decisions made following test results [3, 4]. There has been significant debate about whether some of these tests are 'fit for purpose', for example in the UK, the widespread use of rapid antigen-based lateral flow devices (LFDs) has raised concerns because their sensitivity has been shown to drop dramatically (from 73% to 48.8%) when carried out by non-trained healthcare workers [5-7].

In an attempt to provide greater clarity on the minimum acceptable and desirable characteristics for rapid, point-of-care (PoC) tests for SARS-CoV-2, the World Health Organisation (WHO) and the UK Medicines and Healthcare Products Regulatory Agency (MHRA) developed and published target product profiles (TPPs) for novel tests in this space [8]. However, given the vast number of use cases for PoC tests for SARS-CoV-2 and the ongoing need to provide guidance throughout the UK pandemic, the TPPs were reasonably generic. Further work is required to understand the exact role of these tests within specific settings and the potential downstream consequences of testing [9].

Ambulance services have previously been highlighted as potential settings where rapid PoC testing, including for SARS-CoV-2, could add value for patients and the healthcare system, as they are frequently the first point of contact with healthcare services during an emergency [10-12]. However, due to the unique environment, including reliance upon a dispersed workforce, operating under time pressures and with limited equipment [13], it is necessary to identify setting-specific clinical scenarios where testing could effectively integrate into ambulance care processes. At the time of survey dissemination, at the height of the COVID-19 lockdown in the UK, some ambulance service trusts had begun to trial the use of PoC SARS-CoV-2 testing, but this was not widely performed. The objective of this body of work was to

For peer review only

generate evidence and support decision making on the integration of SARS-CoV-2 PoC tests using a mixed-methodology approach to elicit views from ambulance service staff about factors that would facilitate or act as a barrier to PoC testing for SARS-CoV-2 in this setting. Collecting feedback and insights from end users of PoC devices would enable decision makers to determine the feasibility and expected use cases in an ambulance setting, and work to mitigate any potential barriers to adoption to ensure the greatest added value to healthcare is achieved.

Methods

Study design

This study consisted of two surveys, each following a mixed-methodology approach, designed to capture perceptions on feasibility and prioritisation of potential clinical scenarios where PoC SARS-CoV-2 tests could add value to healthcare. The surveys were developed by test evaluation methodologists (KG, TH, AW, MM and AJA), who are all experienced in qualitative methods for diagnostic evaluation, and were constructed using the online survey tool SurveyMonkey [14]. No questions were mandatory on either survey. No sensitive or patient identifiable data were included.

Respondents to Survey 1 were invited to complete Survey 2.

- Survey 1 [Supplemental materials 1] was launched on 3rd December 2020 and was open to responses until 11th January 2021. The reach of the survey is unknown due to the multiple methods of dissemination, however 212 ambulance service respondents started the survey. Survey 1 aimed to identify key facilitators and barriers to the adoption of PoC SARS-CoV-2 tests in ambulances, with a focus on prioritising the key clinical scenarios where the tests could add value alongside the practical aspects that could affect adoption (environmental factors, test turnaround, usability).
- Survey 2 (follow-up) [Supplemental materials 2] was disseminated to interested parties on 8th February 2021 and was open to responses until 22nd February 2021. 50 respondents to Survey 1 were sent Survey 2 via email. 26/50 (52%) respondents completed Survey 2. This survey aimed to identify the most acceptable diagnostic accuracy requirements of rapid tests specifically to meet the prioritised clinical scenarios that were identified in Survey 1.

Setting

The surveys included in this body of work were conducted at the height of the UK pandemic lockdown period when daily cases were at the highest levels seen in the UK since wide-scale reporting began[15]. Some NHS hospital Trusts reported 100% capacity of critical care beds at this time and the matter was discussed extensively in media and during parliamentary sessions[16]. It is important to highlight this context in mind as it reflects the urgency at which

For peer review only

the work was performed and affected the capacity for participants to respond to the survey, the content of the survey responses and the concerns raised at this time.

Dissemination & participant selection

Participants were selected by purposive sampling with additional snowballing, targeting ambulance service staff at all levels to gain breadth of viewpoints across the service. The primary questionnaire was disseminated via email by Ambulance Trust Research and Development (R&D) teams as well as through promotion of the survey via email (JM) to professionals involved in rapid testing in the acute sector. Survey 2 was disseminated via email directly to those respondents to Survey 1 who had stated that they would be interested in providing additional input through follow-up questionnaire. Participation was voluntary and anonymised.

Purpose & structure

Survey 1 [Supplemental materials 1] was structured with a mixture of multiple choice, Likert and free text answers to capture the following:

1) Demographics, exposure/familiarity with SARS-CoV-2 tests.
2) Desired test characteristics in general (including accuracy, ease of use, time to result (TTR)).
3) Potential clinical scenarios where PoC testing for SARS-CoV-2 testing could add value
4) Feasibility of integrating PoC tests into current processes and patient pathways.

Survey 1 included two open-ended questions: 'What do you think the main concerns of adding a test on board the ambulance would be?' and 'Is there anything else you feel is important to tell us about testing in ambulances?'.

In survey 2 [Supplemental materials 2], respondents were asked to state whether three hypothetical tests with sensitivities and specificities of >95%, >80% and <80% respectively would be useful for each of the priority clinical scenarios identified in Survey 1 (a Likert scale from 'Extremely useful' to 'Not useful at all').

To ensure respondents understood complex technical performance terminology such as sensitivity and specificity, multiple choice answers included an example description of the value, e.g. 60% sensitivity (6/10 people with COVID-19 get a positive test result).

Reporting guidelines

The Standards for Reporting Qualitative Research (SRQR) checklist were used to structure the methodology and results of this study alongside the 'CHERRIES' checklist for reporting of online surveys [17, 18].

Data analysis

Quantitative analysis of structured responses Data processing and generation of summary statistics was performed using Microsoft Excel. As all questions were optional, denominators for each question reflect the number of responses to that individual question. Graphics and visuals were produced using the 'CANVA' web-tool suite to display the aggregated results. For questions using a Likert scale, a mean summary score (with standard deviation) was calculated based on the following mapping - Most Important = 2, Very Important = 1, Important = 0, Less Important = -1, Not Important = -2. Final scores were then ranked from highest to lowest.

Thematic analysis of free text responses A Framework Analysis Method[19] was used to organise data from the qualitative, open-ended questions. Thematic analysis aimed to identify common issues and concerns about testing within the ambulance service. Initial coding of the responses were performed independently by two researchers (KG and MM), with a third acting as adjudicator (AJA) to reduce bias and improve consistency. Codes were discussed internally to reach an agreement on definitions, content and to improve validity. Codes were then clustered under four main categories to identify cross-cutting patterns. Final themes and definitions were agreed collaboratively amongst the qualitative researchers.

Patient and public involvement

The National Institute for Health Research (NIHR) Newcastle In Vitro Diagnostics Co-operative (MIC) Insight patient and public involvement and engagement (PPIE) panel were consulted at various stages of the project, from conception to closing. Comments were received on acceptability of questions, length of the survey, and expected impact of participating in this work. Initial comments were gathered from the PPIE panel on questions exploring COVID-19 testing and interpretation of results. Their feedback helped to formulate and refine questions to better facilitate participant understanding. The PPIE panel suggested that additional information on the terms 'sensitivity' and 'specificity' be included in the survey. Additionally, the PPIE leads wrote a lay summary of the project outputs for dissemination via the MIC website and social media.

Results

Demographics

Of the 212 ambulance service respondents, 150 were listed as 'Paramedics' with 55 of those considered 'Experienced paramedics' – with more than 2 years of experience in the role. Other roles included technical, support and call-handling staff.

One hundred and fourteen (55.8%) of the ambulance staff respondents (hereafter referred to as 'respondents') were based in the East of England, with a further 46 (21.7%) based in the Greater London region (Table 1). All regions of England and Northern Ireland were represented by at least one respondent.

For peer review only

Table 1: Summary table of ambulance service responders by region and job role.

Region	Ambulance Service Staff (Total)	Paramedics & Experienced Paramedics	Technicians & Assistants	Other Staff
**East Midlands**	**5**	3	2	0
**East of England**	**114**	72	35	7
**Greater London**	**46**	36	9	1
**North East**	**2**	2	0	0
**North West**	**3**	3	0	0
**Northern Ireland**	**1**	1	0	0
**South East**	**13**	10	3	0
**South West**	**8**	5	3	0
**West Midlands**	**5**	5	0	0
**Yorkshire & the Humber**	**15**	13	2	0
**Total**	**212**	**150**	**54**	**8**

Feasibility of testing

The majority of respondents answering questions on feasibility stated that it would be feasible to test patients for SARS-CoV-2 before transfer to hospital with 143/190 (75.3%) stating 'High' or 'Very high' feasibility, including 22/26 (84.6%) of the respondents who had previously noted prior experience of using lateral flow devices (LFDs) for SARS-CoV-2 or other indications.

Twenty six out of 101 (24.7%) stated they had experience conducting tests for any respiratory infection in a pre-hospital setting, including (LFDs) and oxygen saturation monitors. Additionally, blood glucose monitoring tests were already in use and routinely performed on route to secondary care.

Use cases and sampling method

The top four ranked clinical scenarios where a PoC test for SARS-CoV-2 was deemed to add value, accounted for 92.4% of the total responses to this question [Figure 1]:

1) '*Triaging of patients prior to arrival at secondary care facilities to improve handover*' (83/170, 48.8%);
2) '*Aid decision making on where a patient should be referred to next*' (40/170, 23.5%);
3) '*Risk stratification of patients to determine if a patient can be safety left at home*' (28/170, 16.5%);
4) '*Rationalising PPE use for ambulance service staff*' (6/170, 3.5%).

Other responses were free-text answers including 'all options', 'none of the options' and other niche uses.

Irrespective of other technology considerations, finger prick blood sampling was stated as the optimal sampling technique by one third of the respondents (125/189, 66.1%). Twenty nine

(29/189, 15.3%) and 19 (19/189, 10.1%) respondents preferred nasopharyngeal swabbing and saliva sampling, respectively. Respondents using the ‘other’ option were largely in favour of testing, regardless of the specific method used.

Importance of test characteristics ‘Accuracy’ and ‘Time to result’ of the test were the most important characteristics to the responders with a mean adjusted score of 1.47 (std.dev 0.44) and 1.32 (std.dev 0.35), respectively [Figure 2]. ‘Storage of the kit’ (mean score 0.14) and ‘size of the kit’ (mean score -0.13) were amongst the least important characteristics. Additional comments provided by paramedics included ‘minimisation of staff exposure time’, ‘room temperature storage’ and ‘cost’.

Time to results 88/170 respondents (51.8%) stated a maximum acceptable TTR of 15 minutes or less, with 162/170 (95.3%) selecting 30 minutes or less.

Sensitivity & Specificity The majority of responses required over 80% sensitivity (56/170, 32.9% selected ‘above 80% sensitivity’, 73/170, 42.9% of responders selected ‘above 90% sensitivity’). Similar responses were obtained for minimum specificity requirements (43/170, 24.7% respondents selected ’above 80% specificity’, and 79/170, 46.5% selected ‘above 90% specificity’).

Thematic analysis of qualitative responses A total of 11 subthemes were generated from the 128 responses to the two free-text questions from the primary survey (Survey 1) and categorised into 4 broad themes (Service level implications, Direct impact on patient care, Practical use of the test and Safety) to identify cross-cutting patterns. Subtheme definitions have been includes in table format [Table 2]. Representative quotes in the results have been assigned a numerical identification code (IC) based on [participant number.

Table 2: Summary table of themes for thematic analysis of open-ended responses.

THEME	SUB-THEME	DESCRIPTION	EXAMPLES
Service level implications	misuse of service	Implications for 999 service and unnecessary paramedics call out (Public and other healthcare providers misusing the Ambulance service)	*“The general public will eventually find out and will call 999 just to get a test.”*<br><br>*“People will call 999 just to be tested. Also 111 & GPs will send us just to test. We should only be responding if symptoms suggest it is a 999 emergency”*<br><br>*“distracting from the primary cause for 999 calls”*
Service level implications	workload	Concerns regarding additional workload due to SARS-COV-2 testing	*“something else to do”*<br><br>*“additional call outs from patients and other healthcare organisations as a means of rapid testing”*<br><br>*“the wider ambulance service resource demand”*

Service level implications	manage public perception	Comments on managing public opinion or perception of the use of COVID testing in ambulances	*"Patients have been omitting to tell crews about COVID symptoms as they feel they won't get an ambulance if they tell the truth"*<br><br>*"people think we do it anyway"*
Impact on patient care	consequences to care	Impact on the system and on patient care (e.g. use of PPE)	*"False positives/negatives giving false sense of security / resulting in incorrect decisions being made based on that information"*<br><br>*"Culture of wearing PPE would relax"*
Impact on patient care	use case to inform clinical decisions	Concerns around how/who/when/why to test, and effect or impact of the result on decision making or conveyance	*"Does the result really change the outcome especially if leaving someone at home?"*<br><br>*"Why do we need to test? We treat for what we see. If someone is very ill they go to hospital, if they aren't they could stay at home and be referred on. A COVID diagnosis doesn't make a difference"*
Impact on patient care	acceptance	Test acceptance and 'trust' on results	*"Patients have been omitting to tell crews about COVID symptoms as they feel they won't get an ambulance if they tell the truth"*
Impact on patient care	time implications	Delay to service or conveyance due to testing or time to results	*"ensuring it does not delay transfer, especially in time critical patients"*<br><br>*"delay will increase the time the patient will be with the driver and thus may affect the Key performance Indicators"*<br><br>*"increased job cycle times"*<br><br>*"delaying treatment or transport due to waiting for results"*<br><br>*"delays on scene"*<br><br>*"Some people wear level 3 PPE for any COVID +ve patient, meaning if they tested +ve they would then delay things further by going to change PPE"*

Practical use of the test	test feasibility, correct use, and integration with ambulance setting	Usability issues, additional skillset required, appropriate staff training for use of the test and integration with ambulance setting. Storage, portability and stocking of the tests in the ambulance and a paramedic kit	*"How it will be carried - it needs to be introduced into standard response bags that are taken to every patient, similar to our blood sugar testing kits. No one will want to go back to the ambulance to collect a large, bulky box to do it"*<br><br>*"kits won't be stored correctly due to fluctuating temperatures of a vehicle outside in all weathers"*<br><br>*"keeping in a sterile place"*<br><br>*"Quantity & Supply in order to test volume of patients in a shift (~5-9 per 12 hour shift). Availability of tests for relatives"*<br><br>*"a naso swab could be difficult in a moving vehicle, test would need to be carried out prior to transport"*
Practical use of the test	integration into pathways	Test accuracy and time to results	*"rely on the test having very high specificity and less important but high sensitivity"*<br><br>*"That it is not accurate enough but hospitals will begin to rely on it and not follow up test"*<br><br>*"If it is not specific enough it could lead to clinicians making inaccurate decisions about patient care"*
Safety	risk exposure for staff members	Concerns and comments on risk of exposure to staff	*"Increased risk to staff carrying out [the] test"*<br><br>*"chance of using AGP's then we could use a L3 mask instead"*<br><br>*"That we will increase our on scene time with a positive patient"*
Safety	Staff reassurance	Staff reassurances	*"would assist in reassurance for crews"*

<u>Category 1 - Service level implications</u>

Misuse of service

The major concern from respondents for the roll out of an ambulance based rapid SARS-CoV-2 test was the misuse of the service by the public, appearing in almost half of the responses – *"The general public will eventually find out and will call 999 just to get a test." [IC7]* This concern also expanded into other service providers too – *"People will call 999 just to be tested. Also 111 & GPs will send us just to test. We should only be responding if symptoms suggest it is a 999 emergency" [IC74]*. Many felt that offering SARS-COV-2 testing would result in increased call volume and a resulting increase in call-outs to patients in a non-critical situation *"distracting from the primary cause for 999 calls" [IC158]*. These concerns were commonly cited with fear of increased workload for ambulance service staff.

Workload

The potential for additional work to be added in the form of processing and performing tests for SARS-COV-2 prompted some strong, negative responses commonly paired with concerns involving the misuse of the ambulance service. Fundamentally, some service staff considered the SARS-COV-2 test *"something else to do" [IC70]* in an otherwise busy and time critical role. Concerns were raised about *"additional call outs from patients and other healthcare organisations as a means of rapid testing" [IC81]* leading to an impact on the efficiency *and "the wider ambulance service resource demand" [IC55]*.

Public expectation

Responses reflected upon previous experience, with cases of the public not declaring disease or symptoms to avoid incorrect perceived stigma or reduced care – *"Patients have been omitting to tell crews about COVID symptoms as they feel they won't get an ambulance if they tell the truth" [IC42]*. Further concerns stemmed from the overarching belief that the general public may use the ambulance service as a more convenient way to get a SARS-COV-2 test – *"Do not advertise to the public" [IC52], "If testing were to be carried out by ambulances, attempts should be made to keep this away from public knowledge" [IC97]*. Other respondents suggested that the general public already believes that the ambulance services provides routine SARS-COV-2 testing *"people think we do it anyway" [IC178]*, a concern that has already been noted as an issue for some ambulance trusts in previous waves [20].

Category 2 - Impact on patient care

Consequences to care

Confidence in rapid SARS-COV-2 tests appeared to be low with concerns over increased harm for patients and staff associated with erroneous results *"False positives/negatives giving false sense of security / resulting in incorrect decisions being made based on that information" [IC91].* Additional concerns were raised regarding staff adherence to PPE requirements - *"Culture of wearing PPE would relax" [IC108]*.

Informing clinical decisions

Overall, the added value of testing was questioned. Comments on who, when, where or why to test were captured, including - *"Does the result really change the outcome especially if leaving someone at home?" [IC28], "Why do we need to test? We treat for what we see. If someone is very ill they go to hospital, if they aren't they could stay at home and be referred on. A COVID diagnosis doesn't make a difference" [IC94].* Multiple responses inferred that testing would not change decision making, referring to the needs of the patient above or beyond their SARS-COV-2 status – *"Value in most patients limited, other clinical indicators rule COVID in or out, and if they are unable to cope at home they have to be conveyed" [IC145]*.

Acceptance

Few respondents noted concerns with acceptance of testing or acceptability of test results, but did reflect serious implications in the potential rollout of the test, especially regarding informed consent and other ethical considerations when weighed against the inherent risk of a pandemic situation. Representative comments included *"The public refusing to take it"*

[IC42] and *"comfort for patients" [IC64]* highlighting concerns over the public reaction to testing.

Time implications

While frequently noted alongside concerns surrounding 'misuse of service' and 'consequences to care', comments directly regarding delays to conveyance as a result of incorporating a SARS-COV-2 test or other knock-on delays to the service were significant. Some responses followed a patient-centric reasoning - *"ensuring it does not delay transfer, especially in time critical patients" [IC14]*, while other comments were concerned about the implications such a delay would have on the ambulance service as a whole – *"delay will increase the time the patient will be with the driver and thus may affect the Key performance Indicator (KPI)" [IC44]* and *"increased job cycle times" [IC162]*. Time to result was a key concern amongst paramedics. This was reflected in the open-response answers *as "delaying treatment or transport due to waiting for results" [IC122], "delays on scene" [IC113]* and *"delaying transport while awaiting results" [IC83]*. Additionally, the use of PPE when working with COVID-19 positive patients was noted as having time implications *"Some people wear level 3 RPE for any covid +ve patient, meaning if they tested +ve they would then delay things further by going to change PPE" [IC161]* implying additional time implications beyond the process of administering the test itself and awaiting results although current guidance considers the use of PPE mandatory in any confirmed or suspected COVID-19 case [21].

Category 3 - Practical use of the test

Test feasibility, correct use, and integration with ambulance setting

Non-accuracy and time-to-result related test characteristics and associated properties such as usability were cited, along with additional direct implications regarding staff training. Size, storage and physical characteristics were commonly referenced in responses - *"How it will be carried - it needs to be introduced into standard response bags that are taken to every patient, similar to our blood sugar testing kits. No one will want to go back to the ambulance to collect a large, bulky box to do it" [IC34]*. Further elaboration regarding the limitations posed by the settings within an ambulance were raised – *"kits won't be stored correctly due to fluctuating temperatures of a vehicle outside in all weathers" [IC36]* and *"keeping in a sterile place" [IC40].* Issues regarding physical constraints of an ambulance were also invoked though responses *"Quantity & Supply in order to test volume of patients in a shift (~5-9 per 12 hour shift). Availability of tests for relatives" [IC3]* and the ability of users to actually perform the test in such conditions – *"a naso swab could be difficult in a moving vehicle, test would need to be carried out prior to transport" [IC54].*

Integration into pathways

Test characteristics such as optimum test accuracy and time to results in order to inform decision making were quoted in a larger number of responses, frequently alongside comments on 'consequences to care' and 'use case to inform clinical decisions'. Aspects of test accuracy were mentioned, either as requirements for a test to be worthwhile - *"rely on the test having very high specificity and less important but high sensitivity" [IC13],* or as a

concern of poor accuracy leading to inappropriate clinical decision making – *"That it is not accurate enough but hospitals will begin to rely on it and not follow up test" [IC101], "If it is not specific enough it could lead to clinicians making inaccurate decisions about patient care" [IC107].*

Category 4 - Safety

Risk exposure for staff members

Fear of additional harm as a result of testing included not only breaches in personal protective equipment (PPE) as a result of actually conducting the test *"Increased risk to staff carrying out [the] test" [IC55], "chance of using AGP's then we could use a L3 mask instead" [IC129]* and additionally the increased time required during call outs whilst spent conducting the test *"That we will increase our on scene time with a positive patient" [IC36].*

Staff reassurance

The issue of patient and staff reassurance was covered in a small number of responses - *"would assist in reassurance for crews" [IC30].*

Follow up Survey 2 - Optimal test characteristics for prioritised use cases

Optimal test characteristics for identified clinical scenarios

Respondents were positive about the most accurate hypothetical test (greater than 95% sensitivity and specificity) for use as a decision aid for referral or for triaging of patients prior to arrival at secondary care facilities, but were critical of any test regardless of accuracy being utilised to inform the use of PPE for ambulance staff [Figure 3].

Triaging of patients prior to arrival at a secondary care facility was the use case with the most positive responses based on the hypothetical tests presented, with both tests 'over 95% accurate' and tests 'over 80% accurate' receiving an average rating of 'Very useful'.

The free-text portion of the follow-up survey was frequently used by respondents to support their answers. The main comments included issues with current ambulance staff compliance with PPE requirements and worry that a negative test may lead to lower compliance with PPE requirements. Some noted that rather than relying on a COVID-19 test, ambulance staff use other clinical indicators to influence decision making, particularly when determining if a patient should be conveyed or kept at home based upon standard indicators of illness severity such as pulse and oxygen saturations.

Discussion

Our results suggest that PoC testing for SARS-CoV-2 within ambulances could add value in particular clinical scenarios. However only an accurate, rapid test would provide the information required to inform clinical decision making. Confidence in the feasibility of rolling out such a test appears limited, an issue which has also been highlighted in other clinical settings [11]. Finger prick blood tests were preferred over other methods, perhaps supported

by respondent's prior experience with blood-glucose tests as highlighted in initial scoping questions on prior experience with PoC testing. Small differences in the operation and use of similar PoC tests mean that training and competency requirements would still be necessary and bring with them additional considerations [22].

Generally, the most popular use cases for testing focussed on patient triage, however, the variation in decision making regarding conveyance to hospital is significant between individual ambulance trusts, and test deployment and response is likely to also vary [23]. Compared to Protocol 36-based identification of COVID-19 patients presenting to ambulance services, on-board LFDs may offer a greater positive predictive value and influence more appropriate decision-making on triage and conveyance [24, 25]. Greater evidence generation into situational use of SARS-COV-2 diagnostics may reveal patient sub-groups wherein a rapid test influencing the decision making and conveyance of the patient may improve care without substantially impacting the workload and effectiveness of the ambulance service, but significant concerns raised by ambulance service staff would need to be addressed to ensure adherence.

Pragmatically, the TTR of lateral flow devices for SARS-CoV-2 are typically in the range of 15-30 minutes, fitting with the desired characteristics for paramedics as well as national guidance on reducing hospital admission delays [26]. However, the accuracy they achieve in practice is less than the sensitivity and specificity preferred by respondents to facilitate decision making. The gain in accuracy from typical molecular assays may not have a TTR within the 30 minute window desired by the majority of the respondents. However some molecular assays are available with faster TTR which could be evaluated in this setting [27, 28]. An acceptable TTR alone may not alleviate the main concerns raised by ambulance service staff around misuse of the service, additional training requirements and capacity. Recent publication of service evaluation results for on-board lateral flow device use by paramedics has offered promising results on the accuracy of some LFDs in an in-motion ambulance setting (sensitivity 78%, specificity 100%, TTR 10 mins) when compared to subsequent PCR testing [25].

Misuse of service leading to increased workload is clearly an important concern, although there is no evidence yet that members of the public have called emergency services to specifically obtain a test. The overwhelming volume of responses with these concerns, from geographically diverse regions suggests that these views are shared across many ambulance trusts around the UK. This may have been a reflection of the pandemic situation during the conducting of the survey, where ambulance services were experiencing particularly high handover times and some Accident and Emergency departments operating at capacity [29]. It's possible that issues and concerns regarding misuse of the service for the sole purpose of obtaining SARS-CoV-2 tests has been mitigated by the subsequent (5 April 2021) UK government's announcement to make rapid testing available to everyone in England twice weekly [30].

Overall, the results indicate that ambulance staff are prepared to conduct rapid SARS-CoV-2 testing only if they feel it would add value in making early clinical care decisions, indeed some Ambulance Services trialled the use of on-board lateral flow tests for SARS-CoV-2 at the time this survey work was conducted [25, 31]. There were multiple concerns from respondents

For peer review only

including the impact of additional testing on speed of care delivery and overall service performance as well as the practical constraints of on-board testing and accuracy requirements. Further research is required to characterise and address these concerns before PoC testing for SARS-CoV-2 can be widely adopted into the ambulance service.

Limitations

Limited piloting of the surveys was performed due to the short timespan available to develop, disseminate, collect and analyse results, whilst the short interval of data capture (03/12/2020 – 11/01/2021) limited the reach and coverage of the survey. Due to the nature of dissemination, there was wide regional disparity in respondents, with the majority of participants located in the East of England and Greater London, where ambulance waiting times and emergency department capacities were stretched during the period of data collection. The collection of data from all members of the ambulance service, while useful at the time of collection, may mean that applicability of some responses to a first-line use cases in ambulances may not be coming from first-hand experience in that setting. The majority of respondents were frontline ambulance staff and paramedics and thus were largely representative of the intended population.

Due to the online interface and open availability, the role and positions of respondents and their understanding of test performance descriptions (i.e. sensitivity and specificity) could not be verified. Descriptions and summaries of these words and phrases were included in the survey to ensure understanding. It was evident that some Trusts had been trialling SARS-CoV-2 PoC testing, while others had refrained, meaning that prior experience with such tests was very variable amongst respondents. The dissemination of the survey primarily through ambulance trust R&D teams and direct contact with respondents may mitigate concerns over false data to an extent.

None of the questions in the surveys were compulsory and most answers provided some free text capacity, those with stronger positive or negative opinions on the subject matter may have been more driven to provide their opinions, introducing a degree of selection bias in participants.

The separation of the questions into two surveys may have introduced bias. Survey 2 had a significantly lower response rate than Survey 1 and was only disseminated to those respondents from Survey 1 who had agreed to being contacted again.

While some respondents noted experience of using SARS-CoV-2 LFDs in an ambulance setting through trials, there were not enough of these respondents to allow for sub-group analysis of those with experience of using SARS-CoV-2 LFDs, and those without.

List of abbreviations:

CE-IVD - Conformitè Europëenne, In-Vitro Diagnostics

CONDOR - The COVID-19 National DiagnOstic Research and Evaluation Platform

For peer review only

IC – Identification Codes

IVD - In-Vitro Diagnostics

LFD - Lateral flow device

MHRA - UK Medicines and Healthcare Products Regulatory Agency

MIC – MedTech In Vitro Diagnostics Cooperative

NIHR - National Institute for Health Research

PoC – Point of care

PPE - Personal Protective Equipment

PPIE – Patient and Public Involvement & Engagement

R&D - Research and Development

SRQR – Standards for Reporting Qualitative Research

TPP – Target Product Profiles

TTR – Time to results

WHO – World Health Organisation

Declarations:

Ethics approval statement

This work was registered as a service evaluation in Newcastle upon Tyne Hospitals NHS Foundation Trust (project ID: 10641) and granted Caldicott approval (ID: 7867).

Consent for publication

Consent was obtained from participants after they read the information on the cover page of the survey and agreed to participate by clicking through to the survey questions on the following pages.

Data sharing statement

Anonymised data used in this publication is available from the corresponding author upon reasonable request

Competing interests

The authors declare no conflict of interest pertaining to this body of work.

For peer review only

Funding

This study was part of the CONDOR platform (condor-platform.org), which is funded by the UKRI, Asthma UK and the British Lung Foundation [COV0051]. KG, AW, TH, DAP and AJA are supported by the National Institute for Health Research (NIHR) Newcastle In Vitro Diagnostics Co-operative (http://www.newcastle.mic.nihr.ac.uk/) [MIC-2016-014]. MaM is supported by the NIHR London In Vitro Diagnostics Co-operative (https://london.ivd.nihr.ac.uk/) [MIC-2016-008]. BS is part-funded as an Associate Director of the NIHR Leeds In Vitro Diagnostics Co-operative (https://www.leedsmic.nihr.ac.uk/) [MIC-2016-015]. The funders had no role in study design, data collection and analysis, decision to publish, or preparation of the manuscript.

Contributorship statement

AJA and JM conceived the study and design. KG, AW, TH and MAM contributed to the study design. KG developed the surveys. AJA and MAM contributed to survey development. MAM and JM piloted the surveys. JM, KG and AJA disseminated the survey to Trust R&D teams. KG analysed quantitative data. KG, AJA and MAM analysed free-text responses and performed thematic analysis of qualitative responses. TH completed checklist of qualitative reporting. KG and AJA produced the figures and tables. KG and AJA wrote initial manuscript drafts. BS, LS, CP and KD revised and edited the manuscript. All authors reviewed and agreed with the results and conclusions of this manuscript.

Acknowledgements

The authors would like to thank Graham McClelland for early discussion and scoping for this project, as well as all of the participants who contributed their time and expertise in completing the surveys. The authors are grateful for the input and feedback provided by the MIC Insight Panel and other PPIE members. The authors also acknowledge the support and contributions of the NIHR and CONDOR steering group. Additional members of the CONDOR Steering group include: Dr Emily Adams, Professor Richard Body, Dr Julian Braybrook, Professor Peter Buckle, Professor Paul Dark, Dr Kerrie Davis, Dr Eloïse Cook, Professor Adam Gordon, Mrs Anna Halstead, Dr Charlotte Harden, Dr Colette Inkson, Professor Gail Hayward, Ms Naoko Jones, Professor Dan Lasserson, Dr Joseph Lee, Professor Andrew Lewington, Mrs Mary Logan, Dr Massimo Micocci, Dr Brian Nicholson, Professor Rafael Perera-Salazar, Mr Graham Prestwich, Dr D. Ashley Price, Dr Charles Reynard, Dr Beverley Riley, Professor John Simpson, Mrs Valerie Tate, Dr Philip Turner, Professor Mark Wilcox and Dr Melody Zhifang.

The views expressed in this manuscript are those of the authors and not necessarily those of the NIHR or the Department of Health and Social Care.

References

For peer review only

1. Dinnes, J., et al., *Rapid, point-of-care antigen and molecular-based tests for diagnosis of SARS-CoV-2 infection.* Cochrane Database Syst Rev, 2020. **8**: p. CD013705.
2. FindDX. *FindDX SARS-CoV-2 diagnostic pipeline*. 2021 [cited 2021; Available from: https://www.finddx.org/covid-19/pipeline/.
3. Bossuyt, P.M., et al., *Comparative accuracy: assessing new tests against existing diagnostic pathways.* BMJ, 2006. **332**(7549): p. 1089-92.
4. Ferrante di Ruffano, L., et al., *Assessing the value of diagnostic tests: a framework for designing and evaluating trials.* BMJ, 2012. **344**: p. e686.
5. Garcia-Finana, M., et al., *Performance of the Innova SARS-CoV-2 antigen rapid lateral flow test in the Liverpool asymptomatic testing pilot: population based cohort study.* BMJ, 2021. **374**: p. n1637.
6. Joint PHE Porton Down & University of Oxford SARS-CoV-2 test development and validation cell, *Rapid evalutaion of Lateral Flow Viral Antigen detection devices (LFDs) for mass community testing*. 2021: Oxford University.
7. University of Liverpool, *Innova Lateral Flow SARS-CoV-2 Antigen test accuracy in Liverpool Pilot: preliminary data.* 2020.
8. Medicines & Healthcare products Regulatory Agency, *Target Product Profile: antibody tests to help determine if people have recent infection to SARS-CoV-2: Version 2*. 2021.
9. Graziadio, S., et al., *How to Ease the Pain of Taking a Diagnostic Point of Care Test to the Market: A Framework for Evidence Development.* Micromachines (Basel), 2020. **11**(3).
10. CONDOR steering group. *CONDOR: COVID-19 National DiagnOstic Research and Evaluation Platform*. 2021; Available from: https://www.condor-platform.org/.
11. Graziadio, S., et al., *Unmet clinical needs for COVID-19 tests in UK health and social care settings.* PLoS One, 2020. **15**(11): p. e0242125.
12. Nicholson, B.D., et al., *Rapid community point-of-care testing for COVID-19 (RAPTOR-C19): protocol for a platform diagnostic study.* Diagn Progn Res, 2021. **5**(1): p. 4.
13. Burges Watson, D.L., et al., *Evidence from the scene: paramedic perspectives on involvement in out-of-hospital research.* Ann Emerg Med, 2012. **60**(5): p. 641-50.
14. SurveyMonkey Inc.; Available from: www.surveymonkey.com.
15. Ritchie H, M.E., et al. *Coronavirus Pandemic (COVID-19)*. 2020; Available from: https://ourworldindata.org/coronavirus/country/united-kingdom#how-many-tests-are-performed-each-day.
16. Parliament, U., *COVID-19: NHS Bed Capacity*. 2021.
17. Eysenbach, G., *Improving the quality of Web surveys: the Checklist for Reporting Results of Internet E-Surveys (CHERRIES).* J Med Internet Res, 2004. **6**(3): p. e34.
18. O'Brien, B.C., et al., *Standards for reporting qualitative research: a synthesis of recommendations.* Acad Med, 2014. **89**(9): p. 1245-51.
19. Ritchie, J.L., J; Nicholls, C.M; Ormston, R;, *Qualitative research practice: A guide for social science students and researchers*. 2nd Edition ed. 2013: SAGE Publishing.
20. Association of ambulance chief executives.; Available from: https://aace.org.uk/wp-content/uploads/2020/10/Integrated-Urgent-and-Emergency-Care-Covid-19-Case-Study-240920.pdf.
21. Public Health England. 29/01/2021]; Available from: https://www.gov.uk/government/publications/covid-19-guidance-for-ambulance-trusts/covid-19-guidance-for-ambulance-trusts#personal-protective-equipment.
22. Blanchard, I.E., et al., *Community paramedic point of care testing: validity and usability of two commercially available devices.* BMC Emerg Med, 2019. **19**(1): p. 30.
23. O'Cathain, A., et al., in *Understanding variation in ambulance service non-conveyance rates: a mixed methods study*. 2018: Southampton (UK).

For peer review only

24. Fitzpatrick, D., et al., *Epidemiology of emergency ambulance service calls related to COVID-19 in Scotland: a national record linkage study.* Scand J Trauma Resusc Emerg Med, 2022. **30**(1): p. 9.

25. Richards, A., et al., *Evaluation of pre-hospital COVID-19 rapid antigen tests by paramedics and their use in a direct admission pathway.* J Infect, 2022. **85**(3): p. e53-e55.

26. Lord Carter, *Operational productivity and performance in English NHS Ambulance Trusts: Unwarranted variations*. 2018.

27. Dinnes, J., et al., *Rapid, point-of-care antigen and molecular-based tests for diagnosis of SARS-CoV-2 infection.* Cochrane Database Syst Rev, 2021. **3**: p. CD013705.

28. Schildgen, V., et al., *Limits and Opportunities of SARS-CoV-2 Antigen Rapid Tests: An Experienced-Based Perspective.* Pathogens, 2021. **10**(1).

29. England, N., *Urgent and Emergency care daily situation reports 2020-21*, N. England, Editor. 2021.

30. UK government. *Twice weekly rapid testing to be available to everyone in England*. 2021; Available from: https://www.gov.uk/government/news/twice-weekly-rapid-testing-to-be-available-to-everyone-in-england.

31. Service;, S.C.A. *SCAS tests patients for COVI-19 before transfer to hospital in pioneering study.* 2021; Available from: https://www.scas.nhs.uk/scas-tests-patients-for-covid-19-before-transfer-to-hospital-in-pioneering-study/.

Figure 1: Summary of use cases for rapid SARS-COV-2 testing in ambulances

170 respondents stated their preferred use cases for a rapid SARS-COV-2 test in the ambulance service as displayed in this stacked chart. The length of the bar represents the number of respondents providing that use case, with the colours representing the categories of ambulance service staff. A call-out text box displays the main quotes from respondents that noted 'other' as their preferred use case.

Figure 2: Summary scores for most important test characteristics for SARS-COV-2 testing in an ambulance setting

Summary scores for the Likert ranking of test characteristics for a rapid SARS-COV-2 test in the ambulance service are displayed in this chart. Likert scores ranged from -2 [Not important] to +2 [Most important]. Mean score and standard deviation of the results are displayed in the figure.

Figure 3: Summary scores for perceived usefulness of hypothetical SARS-COV-2 tests for top use cases identified though survey.

Summary scores for the Likert ranking of use cases and accuracy statistics for a rapid SARS-COV-2 test in the ambulance service are displayed in this chart. Likert scores ranged from -2 [Not important] to +2 [Most important]. The length of the bars indicates the mean Likert score, while the colour represents the three hypothetical tests referred to in the figure key.

For peer review only

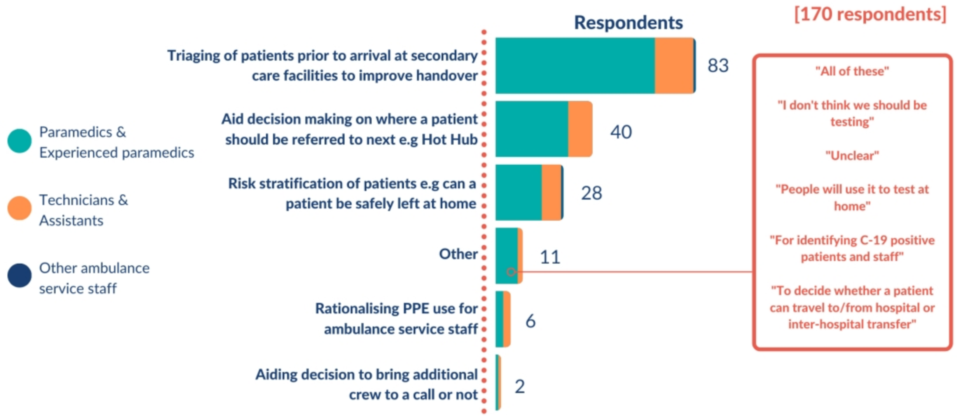

861x484mm (59 x 59 DPI)

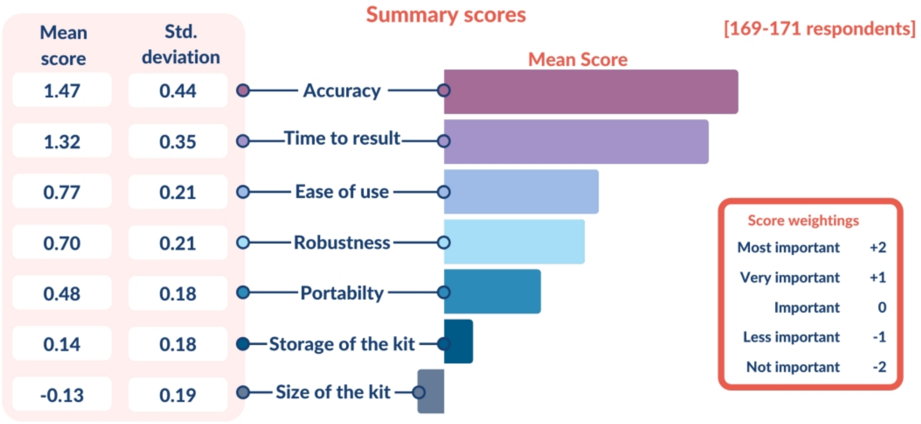

861x484mm (59 x 59 DPI)

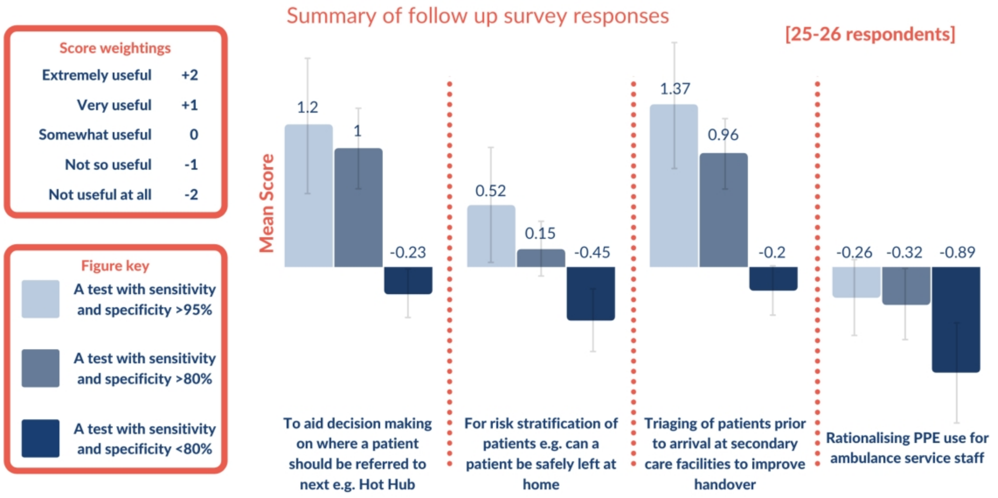

861x484mm (59 x 59 DPI)

Supplementary Material 1: Survey 1 questions

Page 1: Landing page

COVID-19 rapid testing within the Ambulance Service

Dear Participant,

The aim of this survey is to understand the potential role and perceived value of rapid testing within the ambulance service.

You have been invited to participate because you are a health or social care professional in UK.

The survey should take no more than 3 minutes to complete.

No personal or sensitive data is required, and your response is anonymous and will be kept confidential.

Results will be used to inform future research in this area.

If you have any questions or would like to get in touch please do not hesitate to contact: nihr.newcastle.mic@ncl.ac.uk.

By continuing to the next page you will be consenting to participate in this survey.

Thank you for taking part.

Page 2: Participant Information

1. **Which region of the UK do you work in?**

    Scotland

    Northern Ireland

    Wales

    North West England

    North East England

    Yorkshire & The Humber

    West Midlands

    East Midlands

    South West

    South East

    East of England

    Greater London

2. **What is your current job role?**

    Ambulance Service Team – Ambulance care assistant and Patient Transport Service (PTS) driver

    Ambulance Service Team – Call handler/emergency medical dispatcher

    Ambulance Service Team – Emergency care assistant

    Ambulance Service Team – Emergency medical technician

    Ambulance Service Team - Paramedic

    Ambulance Service Team – Experience paramedic

peer review only

Ambulance Service Team – Patient Transport Service (PTS) call handler

Ambulance Service Team – Integrated urgent care/NHS 111

3. **Please use this space to add any other information regarding your role, if applicable (eg. specialism, additional expertise, area of interest)**

   [Free Text]

Page 3: COVID-19 rapid testing within the Ambulance Service

4. **Do you already conduct any tests for respiratory infection in a pre-hospital setting? If yes, please describe the test(s) below:**

   [Free text]

5. **How feasible do you think it would be to test patients for COVID-19 before transfer to hospital?**

   Very high

   High

   Neutral

   Low

   Very Low

   a. **Please add any additional comments on feasibility here:**

   [Free text]

6. **What would be the optimal or ideal sampling technique for a COVID-19 test in an ambulance setting?**

   Nasopharyngeal swab

   Finger prick blood test

   Sputum sample

   Saliva sample

   Other (please specify)

   [Free text]

7. **How helpful do you feel the deployment of rapid COVID-19 testing in an ambulance setting would be?**

   Very helpful

   Quite helpful

   Neither helpful nor unhelpful

   Quite unhelpful

   Very unhelpful

8. **What do you think the main use case for rapid COVID-19 testing in ambulances would be?**

For peer review only

Triaging of patients prior to arrival at secondary care facilities to improve handover and flow

Risk stratification of patients e.g. can a patient be safely left at home

Aid decision making on where the patient should be referred to next e.g. Hot-Hub

Rationalising PPE use for ambulance service staff

Aiding decision to bring additional crew to a call or not

Other (please specify)

[Free Text]

9. What do you think the most important characteristics of a test on board ambulances would be?

Time to result – Most important – very important – important – less important – not important

Accuracy – Most important – very important – important – less important – not important

Ease of use – Most important – very important – important – less important – not important

Robustness – Most important – very important – important – less important – not important

Size of the kit – Most important – very important – important – less important – not important

Storage of the kit – Most important – very important – important – less important – not important

Portability of the kit – Most important – very important – important – less important – not important

Other (please specify) – Most important – very important – important – less important – not important

10. What would be the maximum acceptable time to results (TTR) for an ambulance-based COVID test?

[Slider scale 'Time to results in minutes', Minimum value = 0, Maximum value = 45]

11. What is the minimum sensitivity that a test in ambulances should have?

>60% (at least 6/10 people with COVID will get a positive result)

>70% (at least 7/10 people with COVID will get a positive result)

>80% (at least 8/10 people with COVID will get a positive result)

>90% (at least 9/10 people with COVID will get a positive result)

12. What is the minimum specificity that a test in ambulances should have?

>60% (at least 6/10 people without COVID will get a negative result)

>70% (at least 7/10 people without COVID will get a negative result)

>80% (at least 8/10 people without COVID will get a negative result)

>90% (at least 9/10 people without COVID will get a negative result)

13. What do you think the main concerns of adding a test on board an ambulance would be?

[Free Text]

For peer review only

14. Is there anything else you feel is important to tell us about testing in ambulances?

[Free Text]

15. If you would be willing to speak to us further about COVID-19 testing within the ambulance service, please provide details below

[Name]

[Preferred Email Address]

For peer review only

Supplementary Material 2: Survey 2 questions

Page 1: Landing page

Use cases and diagnostic profiles for COVID-19 rapid testing within the Ambulance Service

Dear Participant,

The aim of this survey is to further understand the potential role for COVID-19 diagnostic tests in different scenarios relevant to the ambulance service.

You have been invited to participate because you are a health or social care professional in UK and have previously provided contact details for us to speak to you further about COVID-19 testing in an ambulance setting.

The follow up survey should take no more than 3 minutes to complete.

No personal or sensitive data is required, and your response is anonymous and will be kept confidential.

Results will be used to inform future research in this area.

If you have any questions or would like to get in touch please do not hesitate to contact: nihr.newcastle.mic@ncl.ac.uk.

By continuing to the next page you will be consenting to participate in this survey.

Thank you for taking part.

Page 2: Use cases and diagnostic profiles for COVID-19 rapid testing within the Ambulance Service

The results of our initial survey highlighted a number of potential use cases that participants felt may be important. In this follow up series of questions we would like to better understand the qualities and characteristics of COVID-19 tests that you feel are necessary for each use case.

Please rate how useful you feel each type of test would be for each of the use cases from 'Extremely useful' to 'Not at all useful'

Sensitivity - The ability of a test to correctly identify a patient with the disease.
[The test is positive when the patient has COVID-19]

Specificity - The ability of a test to correctly identify a patient who does not have the disease.
[The test is negative when the patient does not have COVID-19]

1. **A test with greater than 95% sensitivity and specificity, less than 15 minutes time to results**
    a. Aid decision making on where the patient should be referred to next e.g. Hot-Hub

        Extremely useful – Very useful – Somewhat useful – Not so useful – Not at all useful

    b. Risk stratification of patients e.g. can a patient be safely left at home

        Extremely useful – Very useful – Somewhat useful – Not so useful – Not at all useful

    c. Triaging of patients prior to arrival at secondary care facilities to improve handover and flow

        Extremely useful – Very useful – Somewhat useful – Not so useful – Not at all useful

    d. Rationalising PPE use for ambulance service staff

        Extremely useful – Very useful – Somewhat useful – Not so useful – Not at all useful

- Please provide any additional comments or reasoning here:

  [Free Text]

2. **A test with greater than 80% sensitivity and specificity, less than 15 minutes time to results**
    a. Aid decision making on where the patient should be referred to next e.g. Hot-Hub

Extremely useful – Very useful – Somewhat useful – Not so useful – Not at all useful

b. Risk stratification of patients e.g. can a patient be safely left at home

Extremely useful – Very useful – Somewhat useful – Not so useful – Not at all useful

c. Triaging of patients prior to arrival at secondary care facilities to improve handover and flow

Extremely useful – Very useful – Somewhat useful – Not so useful – Not at all useful

d. Rationalising PPE use for ambulance service staff

Extremely useful – Very useful – Somewhat useful – Not so useful – Not at all useful

- Please provide any additional comments or reasoning here:

  [Free Text]

3. A test with less than 80% sensitivity and specificity, less than 15 minutes time to results

a. Aid decision making on where the patient should be referred to next e.g. Hot-Hub

Extremely useful – Very useful – Somewhat useful – Not so useful – Not at all useful

b. Risk stratification of patients e.g. can a patient be safely left at home

Extremely useful – Very useful – Somewhat useful – Not so useful – Not at all useful

c. Triaging of patients prior to arrival at secondary care facilities to improve handover and flow

Extremely useful – Very useful – Somewhat useful – Not so useful – Not at all useful

d. Rationalising PPE use for ambulance service staff

Extremely useful – Very useful – Somewhat useful – Not so useful – Not at all useful

- Please provide any additional comments or reasoning here:

  [Free Text]

For peer review only

COMPLETION OF THIS CHECKLIST WAS REQUESTED BY REVIEWERS OF THE INITIAL EMJ SUBMISSION

Item Category	Checklist Item	Explanation	Manuscript Response/Description
**Design**	Describe survey design	Describe target population, sample frame. Is the sample a convenience sample? (In "open" surveys this is most likely.)	*The target population was emergency medical staff working in the UK. The survey was 'live' for a set time and was an open survey disseminated by Trust R&D teams and targeted individuals.*
**IRB (Institutional Review Board) approval and informed consent process**	IRB approval	Mention whether the study has been approved by an IRB.	*The project was approved as a service evaluation by NuTH and each participating trust decided internally to disseminate amongst staff.*
	Informed consent	Describe the informed consent process. Where were the participants told the length of time of the survey, which data were stored and where and for how long, who the investigator was, and the purpose of the study?	*The landing page of the survey detailed the purpose of the survey, the estimated length of time of the survey and that sharing personal information was not a required part of the survey. The investigator and contact details were listed on the landing page.*
	Data protection	If any personal information was collected or stored, describe what mechanisms were used to protect unauthorized access.	*All data collected form the survey was downloaded to a password protected computer and stored in a locked room in a Newcastle University building.*
**Development and pre-testing**	Development and testing	State how the survey was developed, including whether the usability and technical functionality of the electronic questionnaire had been tested before fielding the questionnaire.	*The survey was piloted internally within the research group and externally with choice experts. Feedback from the pilot was used to refine and edit the final survey. The results from piloting the survey were not included in the final manuscript.*
**Recruitment process and description of the sample having access to the questionnaire**	Open survey versus closed survey	An "open survey" is a survey open for each visitor of a site, while a closed survey is only open to a sample which the investigator knows (password-protected survey).	*The survey was an open survey in that access to the survey could be made by anyone with the survey-specific link. The link was shared directly with respondents and disseminated by relevant persons.*
	Contact mode	Indicate whether or not the initial contact with the potential participants was made on the Internet. (Investigators may also send out questionnaires by mail and allow for Web-based data entry.)	*The main mode of contact was via email. The link and supporting information was sent directly to participants email addresses.*
	Advertising the survey	How/where was the survey announced or advertised? Some examples are offline media (newspapers), or online (mailing lists – If yes, which ones?) or banner ads (Where were these banner ads posted and what did they look like?). It is important to know the wording of the announcement as it will heavily influence who chooses to participate. Ideally the survey announcement should be published as an appendix.	*The survey was not advertised.*
**Survey administration**	Web/E-mail	State the type of e-survey (eg, one posted on a Web site, or one sent out	*The survey was web-based. There was no manual copying of the*

For peer review only

COMPLETION OF THIS CHECKLIST WAS REQUESTED BY REVIEWERS OF THE INITIAL EMJ SUBMISSION

		through e-mail). If it is an e-mail survey, were the responses entered manually into a database, or was there an automatic method for capturing responses?	*responses into a database. Responses were captured automatically.*
	Context	Describe the Web site (for mailing list/newsgroup) in which the survey was posted. What is the Web site about, who is visiting it, what are visitors normally looking for? Discuss to what degree the content of the Web site could pre-select the sample or influence the results. For example, a survey about vaccination on a anti-immunization Web site will have different results from a Web survey conducted on a government Web site	*The survey was not posted to a website. It was disseminated directly via email.*
	Mandatory/voluntary	Was it a mandatory survey to be filled in by every visitor who wanted to enter the Web site, or was it a voluntary survey?	*The survey was voluntary and no answers were mandatory.*
	Incentives	Were any incentives offered (eg, monetary, prizes, or non-monetary incentives such as an offer to provide the survey results)?	*No incentives were offered.*
	Time/Date	In what timeframe were the data collected?	*The data was collected during the COVID-19 pandemic. Survey 1 was launched on 3rd December 2020 and was open to responses until 11th January 2021. Survey 2 was disseminated to interested parties on 8th February 2021 and was open to responses until 22nd February 2021.*
	Randomization of items or questionnaires	To prevent biases items can be randomized or alternated.	*Randomisation was not performed. Questions generally followed logically and would have been difficult to interpret out of order.*
	Adaptive questioning	Use adaptive questioning (certain items, or only conditionally displayed based on responses to other items) to reduce number and complexity of the questions.	*Questions involving an 'other' or 'NA' option would generate a free-text box where respondents could expand on their answer.*
	Number of Items	What was the number of questionnaire items per page? The number of items is an important factor for the completion rate.	*Survey 1 had 3 questions on page 1, and 12 questions on page 2. Survey 2 had 3 questions on a single page.*
	Number of screens (pages)	Over how many pages was the questionnaire distributed? The number of items is an important factor for the completion rate.	*Survey 1 was composed of 4 pages, 2 of which had questions: Landing page, page 1 'demographics', page 2 'COVID-19 testing', Final page 'Survey Complete'. Survey 2 was composed of 3 pages, 1 of which had questions: Landing page, page 1 'follow up questions', Final page 'Survey Complete'.*
		It is technically possible to do	*The survey software noted complete*

COMPLETION OF THIS CHECKLIST WAS REQUESTED BY REVIEWERS OF THE INITIAL EMJ SUBMISSION

	Completeness check	consistency or completeness checks before the questionnaire is submitted. Was this done, and if "yes", how (usually JAVAScript)? An alternative is to check for completeness after the questionnaire has been submitted (and highlight mandatory items). If this has been done, it should be reported. All items should provide a non-response option such as "not applicable" or "rather not say", and selection of one response option should be enforced.	*response to the survey questions or noted at which page the survey was prematurely ended.*
	Review step	State whether respondents were able to review and change their answers (eg, through a Back button or a Review step which displays a summary of the responses and asks the respondents if they are correct).	*Reponses could be changed at any point during the survey by altering answers or using the 'back' and 'forward' buttons to navigate between pages.*
**Response rates**	Unique site visitor	If you provide view rates or participation rates, you need to define how you determined a unique visitor. There are different techniques available, based on IP addresses or cookies or both.	*Each respondent was assigned a unique numerical ID upon starting the survey.*
	View rate (Ratio of unique survey visitors/unique site visitors)	Requires counting unique visitors to the first page of the survey, divided by the number of unique site visitors (not page views!). It is not unusual to have view rates of less than 0.1 % if the survey is voluntary.	*This information would be misleading. The view rate of the surveys was high based on visitors to the first page. This was because the survey was disseminated via email and those that were interested would have clicked on the survey link, while those who were not interested would not have responded to the email, or clicked the link. The reach of the survey is unknown due to the varied dissemination routes. All visitors to the site were linked directly to the first 'landing page' of the survey.*
	Participation rate (Ratio of unique visitors who agreed to participate/unique first survey page visitors)	Count the unique number of people who filled in the first survey page (or agreed to participate, for example by checking a checkbox), divided by visitors who visit the first page of the survey (or the informed consents page, if present). This can also be called "recruitment" rate.	*As above, this value would be misleading as only those potential participants that were interested in completing the survey would visit the weblink supplied in their email. Based on page views, all visitors clicked through the first page of the survey. The reach of the survey is unknown due to method of dissemination.*
	Completion rate (Ratio of users who finished the survey/users who agreed to participate)	The number of people submitting the last questionnaire page, divided by the number of people who agreed to participate (or submitted the first survey page). This is only relevant if there is a separate "informed consent" page or if the survey goes over several pages. This is a measure for attrition. Note that "completion" can involve leaving questionnaire items blank. This	*For purposes of this checklist - One respondent in survey 1 did not complete any answers, all others did. 211/212 = 99.5%.*<br>*Reach of the survey is unknown.*<br><br>*For survey 2, all respondents who started the survey, finished it. 26/26 = 100%*<br>*However, as noted in the above*

For peer review only

COMPLETION OF THIS CHECKLIST WAS REQUESTED BY REVIEWERS OF THE INITIAL EMJ SUBMISSION

		is not a measure for how completely questionnaires were filled in. (If you need a measure for this, use the word "completeness rate".)	*sections, this number is misleading as survey 2 was emailed to 50 participants, with 26 clicking the link to the survey and participating in it. 26/50 = 52% would be a more accurate reflection of the number of participants who agreed to do the second survey and those who actually participated.*
**Preventing multiple entries from the same individual**	Cookies used	Indicate whether cookies were used to assign a unique user identifier to each client computer. If so, mention the page on which the cookie was set and read, and how long the cookie was valid. Were duplicate entries avoided by preventing users access to the survey twice; or were duplicate database entries having the same user ID eliminated before analysis? In the latter case, which entries were kept for analysis (eg, the first entry or the most recent)?	*Each user was assigned a unique numerical ID upon starting the survey. There were no duplicate IDs noted in the results.*<br>*We are not able to rule out the possibility of respondents completing the survey from multiple devices or browsers as anyone with the relevant weblink could access the survey.*
	IP check	Indicate whether the IP address of the client computer was used to identify potential duplicate entries from the same user. If so, mention the period of time for which no two entries from the same IP address were allowed (eg, 24 hours). Were duplicate entries avoided by preventing users with the same IP address access to the survey twice; or were duplicate database entries having the same IP address within a given period of time eliminated before analysis? If the latter, which entries were kept for analysis (eg, the first entry or the most recent)?	*Each user was assigned a unique numerical ID upon starting the survey. There were no duplicate IDs noted in the results.*<br>*We are not able to rule out the possibility of respondents completing the survey from multiple devices or browsers as anyone with the relevant weblink could access the survey.*
	Log file analysis	Indicate whether other techniques to analyze the log file for identification of multiple entries were used. If so, please describe.	*This was not performed.*
	Registration	In "closed" (non-open) surveys, users need to login first and it is easier to prevent duplicate entries from the same user. Describe how this was done. For example, was the survey never displayed a second time once the user had filled it in, or was the username stored together with the survey results and later eliminated? If the latter, which entries were kept for analysis (eg, the first entry or the most recent)?	*Once a user had completed the survey once, that same user would not be able to complete the survey again.*<br>*Users may have been able to circumvent this by changing browsers or using an alternative device.*<br>*Access to the survey was not password protected.*
**Analysis**	Handling of incomplete questionnaires	Were only completed questionnaires analyzed? Were questionnaires which terminated early (where, for example, users did not go through all questionnaire pages) also analyzed?	*One respondent did not complete any answers. This instance was removed from the final analysis.*<br>*All questions were optional, the figures in the manuscript reflect the number of responses received to*

For peer review only

COMPLETION OF THIS CHECKLIST WAS REQUESTED BY REVIEWERS OF THE INITIAL EMJ SUBMISSION

			each individual question.
	Questionnaires submitted with an atypical timestamp	Some investigators may measure the time people needed to fill in a questionnaire and exclude questionnaires that were submitted too soon. Specify the timeframe that was used as a cut-off point, and describe how this point was determined.	*The survey was relatively short. There was no minimum time limit for responses to be considered valid.*
	Statistical correction	Indicate whether any methods such as weighting of items or propensity scores have been used to adjust for the non-representative sample; if so, please describe the methods.	*Likert scores were used in both surveys with responses weighted as described in the manuscript. Weighting of individual participant's responses above or below others was not performed.*

For peer review only

Standards for Reporting Qualitative Research (SRQR)*

http://www.equator-network.org/reporting-guidelines/srqr/

Title and abstract

	Page/line no(s).
**Title** - Concise description of the nature and topic of the study Identifying the study as qualitative or indicating the approach (e.g., ethnography, grounded theory) or data collection methods (e.g., interview, focus group) is recommended	Page 1/Lines 1-4
**Abstract** - Summary of key elements of the study using the abstract format of the intended publication; typically includes background, purpose, methods, results, and conclusions	Pages 1-2/Lines 21-48

Introduction

**Problem formulation** - Description and significance of the problem/phenomenon studied; review of relevant theory and empirical work; problem statement	Page 3/Lines 65-97
**Purpose or research question** - Purpose of the study and specific objectives or questions	Page 3/Lines 90-97

Methods

**Qualitative approach and research paradigm** - Qualitative approach (e.g., ethnography, grounded theory, case study, phenomenology, narrative research) and guiding theory if appropriate; identifying the research paradigm (e.g., postpositivist, constructivist/ interpretivist) is also recommended; rationale**	Page 4/Lines 102-122
**Researcher characteristics and reflexivity** - Researchers' characteristics that may influence the research, including personal attributes, qualifications/experience, relationship with participants, assumptions, and/or presuppositions; potential or actual interaction between researchers' characteristics and the research questions, approach, methods, results, and/or transferability	Page 4/ Lines 104-107
**Context** - Setting/site and salient contextual factors; rationale**	Page 4/ Lines 124-131
**Sampling strategy** - How and why research participants, documents, or events were selected; criteria for deciding when no further sampling was necessary (e.g., sampling saturation); rationale**	Page 5/Lines 134-141
**Ethical issues pertaining to human subjects** - Documentation of approval by an appropriate ethics review board and participant consent, or explanation for lack thereof; other confidentiality and data security issues	Pages 5 & 16 / Lines 140-141, 479-481
**Data collection methods** - Types of data collected; details of data collection procedures including (as appropriate) start and stop dates of data collection and analysis, iterative process, triangulation of sources/methods, and modification of procedures in response to evolving study findings; rationale**	Pages 4, 5 / Lines 110-122, 144-160

For peer review only

For peer review only - http://bmjopen.bmj.com/site/about/guidelines.xhtml

**Data collection instruments and technologies** - Description of instruments (e.g., interview guides, questionnaires) and devices (e.g., audio recorders) used for data collection; if/how the instrument(s) changed over the course of the study	Page 4 / Lines 104-107
**Units of study** - Number and relevant characteristics of participants, documents, or events included in the study; level of participation (could be reported in results)	Page 5 & 6 / Lines 144-157 & 199-201
**Data processing** - Methods for processing data prior to and during analysis, including transcription, data entry, data management and security, verification of data integrity, data coding, and anonymization/de-identification of excerpts	Page 6 / Lines 168-183
**Data analysis** - Process by which inferences, themes, etc., were identified and developed, including the researchers involved in data analysis; usually references a specific paradigm or approach; rationale**	Page 6 / Lines 168-183
**Techniques to enhance trustworthiness** - Techniques to enhance trustworthiness and credibility of data analysis (e.g., member checking, audit trail, triangulation); rationale**	Page 6 / Lines 178-183

Results/findings

**Synthesis and interpretation** - Main findings (e.g., interpretations, inferences, and themes); might include development of a theory or model, or integration with prior research or theory	Pages 6-13 / Lines 196-375
**Links to empirical data** - Evidence (e.g., quotes, field notes, text excerpts, photographs) to substantiate analytic findings	Pages 8-10 / Table 2 / Line 253

Discussion

**Integration with prior work, implications, transferability, and contribution(s) to the field -** Short summary of main findings; explanation of how findings and conclusions connect to, support, elaborate on, or challenge conclusions of earlier scholarship; discussion of scope of application/generalizability; identification of unique contribution(s) to scholarship in a discipline or field	Pages 13-15 / Lines 377-428
**Limitations** - Trustworthiness and limitations of findings	Page 15 / Lines 429-458

Other

**Conflicts of interest** - Potential sources of influence or perceived influence on study conduct and conclusions; how these were managed	Page 16 / Lines 492-493
**Funding** - Sources of funding and other support; role of funders in data collection, interpretation, and reporting	Page 17 / Lines 494-503

*The authors created the SRQR by searching the literature to identify guidelines, reporting standards, and critical appraisal criteria for qualitative research; reviewing the reference lists of retrieved sources; and contacting experts to gain feedback. The SRQR aims to improve the transparency of all aspects of qualitative research by providing clear standards for reporting qualitative research.

For peer review only - http://bmjopen.bmj.com/site/about/guidelines.xhtml

**The rationale should briefly discuss the justification for choosing that theory, approach, method, or technique rather than other options available, the assumptions and limitations implicit in those choices, and how those choices influence study conclusions and transferability. As appropriate, the rationale for several items might be discussed together.

Reference:
O'Brien BC, Harris IB, Beckman TJ, Reed DA, Cook DA. **Standards for reporting qualitative research: a synthesis of recommendations.** *Academic Medicine*, Vol. 89, No. 9 / Sept 2014 DOI: 10.1097/ACM.0000000000000388

For peer review only
